# Local Electric Fields Originate Unusual Kinetic Isotope Effects in the Hydrogen Abstraction Reactions of the Functionally Analogous P450 and TauD Enzymes

**DOI:** 10.1021/jacs.5c10488

**Published:** 2025-09-17

**Authors:** Surajit Kalita, David Danovich, Sason Shaik

**Affiliations:** Institute of Chemistry, The Hebrew University of Jerusalem, Edmond J. Safra Campus, Givat Ram, Jerusalem 9190401, Israel

## Abstract

Cytochrome P450 enzymes (P450s) and their functionally analogous TauD enzyme (taurine D α-KG-dependent dioxygenase) oxidize alkanes by an initial hydrogen atom abstraction. We use molecular dynamics (MD) simulations and hybrid quantum mechanics/molecular-mechanics (QM/MM) calculations to comprehend the origins of the **counterintuitive kinetic isotope effects** (KIEs), which are exhibited by these two enzyme families. Thus, P450s exhibit low KIE values, which indicate the absence of quantum mechanical tunneling (QMT) contributions, whereas TauD exhibits high KIEs, which apparently include QMT. Furthermore, the calculations show that the P450 protein-folds compensate for the lack of QMT, by reactivity enhancement due to the favorable interactions of the substrate(s) with the corresponding local electric field (LEF) of these enzymes. By contrast, TauD exhibits higher hydrogen abstraction barriers as well as a significant QMT. Thus, the relative reactivities of the two enzyme-types, toward a given substrate, are determined by the larger LEF in P450s vis-à-vis the higher QMT in TauD. The present manuscript provides a comprehensive understanding of the root-causes of the LEF effects of the protein-folds vs the QMT factors, in modulating hydrogen-atom abstraction reactivity, in two distinct mechanistic strategies, for the functionally analogous P450 and TauD enzymes.

## Graphical abstract

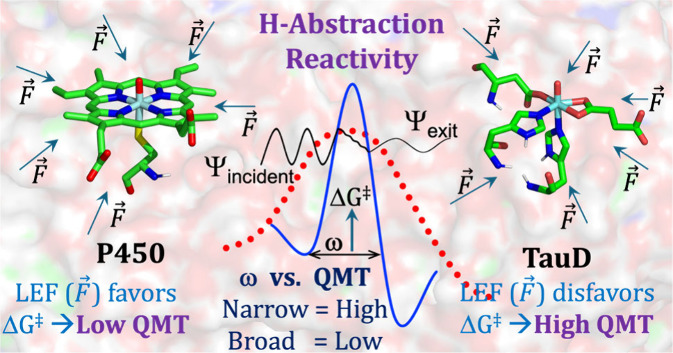

## 1 Introduction

Quantum mechanical tunneling (QMT) is a phenomenon that is rooted in the wave nature of particles and can have significant consequences in chemical reactivity of molecules.^1,2^ In addition to QMT, local electric fields (LEFs) play a crucial role in modulating chemical reactivity in enzymatic reactions. This LEF concept was initially proposed by Warshel^3–5^ and later experimentally verified by the Boxer group using vibrational Stark spectroscopy.^6,7^

In a chemical reaction, atoms/molecules can act wave-like and penetrate below the transition state of the potential energy surface (PES) due to QMT ((a) in Scheme 1). This reduces the actual energy barrier and, consequently, enhances the rate of the reaction vis-à-vis the rate predicted by transition state theory (TST).^1,2^ The QMT effect on reactivity can be experimentally determined using the kinetic isotope effect (KIE), which is defined as the ratio of rate constants for the reactions with the lighter isotope vis-à-vis its heavier isotope.^8,9^ Equation 1 defines this ratio, wherein *k*_H_ and *k*_D_ are the respective rate constants for the H and D abstractions by the oxo-iron species of P450 (Scheme 1b) and TauD (Scheme 1c).

1 $$\mathrm{KIE}=\frac{k_{H}}{k_{D}}$$

**Scheme 1:**
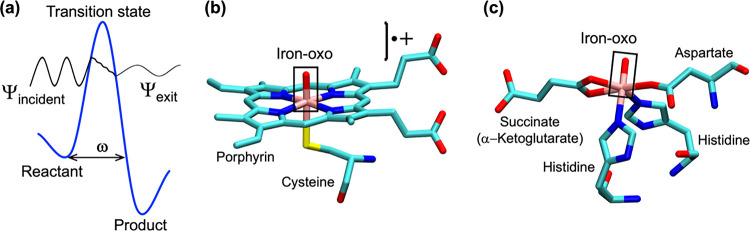
(a) A cartoon illustration of QMT, showing how atomic particles (H/D) tunnel through a barrier of width ω. Ψ_incident_ and Ψ_exit_ represent the wavefunctions of the incident-reactant and -product molecules, respectively. The H/D abstraction reactions transpire by the high-valent oxo-iron active species in (b) and (c): (b) P450 enzymes feature oxo-iron and a porphyrin cation-radical. (c) The oxo-iron species of α-KG-dependent dioxygenase nonheme enzyme (TauD).

Although heavy metal tunneling has gained considerable interest in recent years,^10,11^ the effects of QMT are more pronounced in the lighter hydrogenic atoms.^1,2^ As such, one of the most fundamental chemical reactions, hydrogen atom transfer (HAT),^12^ is expected to be significantly affected by QMT. We note that HAT is used herein as an umbrella term for all reactions involving the transfer of a hydrogen atom, irrespective of the electronic mechanisms, such as proton-coupled electron transfer (PCET), electron transfer followed by proton transfer (ET-PT), and vice versa.^12,13^ HAT events are common in a wide range of organic reactions^14,15^ and are also predominantly found in biologically relevant enzymatic processes.^16,17^ For instance, two such prominent iron-centered enzyme families that perform HAT reactions are (a) heme-containing cytochrome P450 monooxygenases, in Scheme 1b,^18,19^ and (b) the nonheme, α-ketoglutarate (α-KG)-dependent dioxygenases,^20,21^ in Scheme 1c.

Scheme 1b displays the P450 active species, so-called Compound I (Cpd I), wherein the porphyrin (Por) is in a radical-cationic state (Por^•+^). Cpd I is a powerful oxidant, which can be represented using oxidation numbers as Por^•+^Fe^IV^O. P450 enzymes feature also another species, PorFe^IV^O, known as Cpd II, where the porphyrin is a closed shell species. Cpd II is generally a weaker oxidant than Cpd I.^18,19^ Note that the nonheme species in Scheme 1c is a Cpd II-type species, L_4_Fe^IV^O, which lacks the additional oxidation equivalent of Cpd I, and as such, should be less reactive than the P450 Cpd I species.^20,21^

Notably, both enzyme families (heme and nonheme) catalyze several important reactions that are vital for all living organisms, including humans, such as the metabolism of drugs and xenobiotics, biosynthesis of collagen, repair of alkylated DNA, etc.^22–24^ Apart from these two enzyme families, other nonheme enzymes, such as soybean lipoxygenase^25^ and methane monooxygenase,^26^ also perform HAT reactions.

HAT reactions which are catalyzed by these enzymes are generally anticipated to display a large KIE value due to QMT. For example, the experimentally determined KIE for the nonheme taurine: α-KG-dependent dioxygenase (TauD) enzyme, is ∼58.^27^ Similarly, soybean lipoxygenase^25^ and methane monooxygenase^26^ exhibit large KIE values of 81 and 50–100, respectively. In the past, the Jerusalem group has computed the KIE of several HAT reactions of organic molecules, that are catalyzed by heme and nonheme iron-oxo complexes.^28–32^ These computational studies revealed generally moderate or low KIE values for HAT reactions of heme enzymes, in good agreement with experimentally determined values.^33–37^

As such, by contrast to HAT reactions in nonheme enzymes, which exhibit high KIE values, HAT reactions catalyzed by the Cpd I species of P450 enzymes produce low KIE values,^25–37^ typically below 10, with some values approaching the classical limit (<10).^36^ Similarly, for two of the most studied bacterial P450 systems, P450_CAM_ and P450_BM3_, the KIE values are ∼1.23 for camphor^38^ and ∼7.3 for *p*-xylene^39^ as substrates.

By contrast, when the HAT reaction was performed in a solvent using the **synthetic Cpd I** ([Fe^IV^(O)­(TMP^•+^(Cl^–^))]), shown in Scheme 2, the authors reported a large experimental KIE of 47 ± 4, *which obviously involves tunneling*.^28,40,41^

**Scheme 2:**
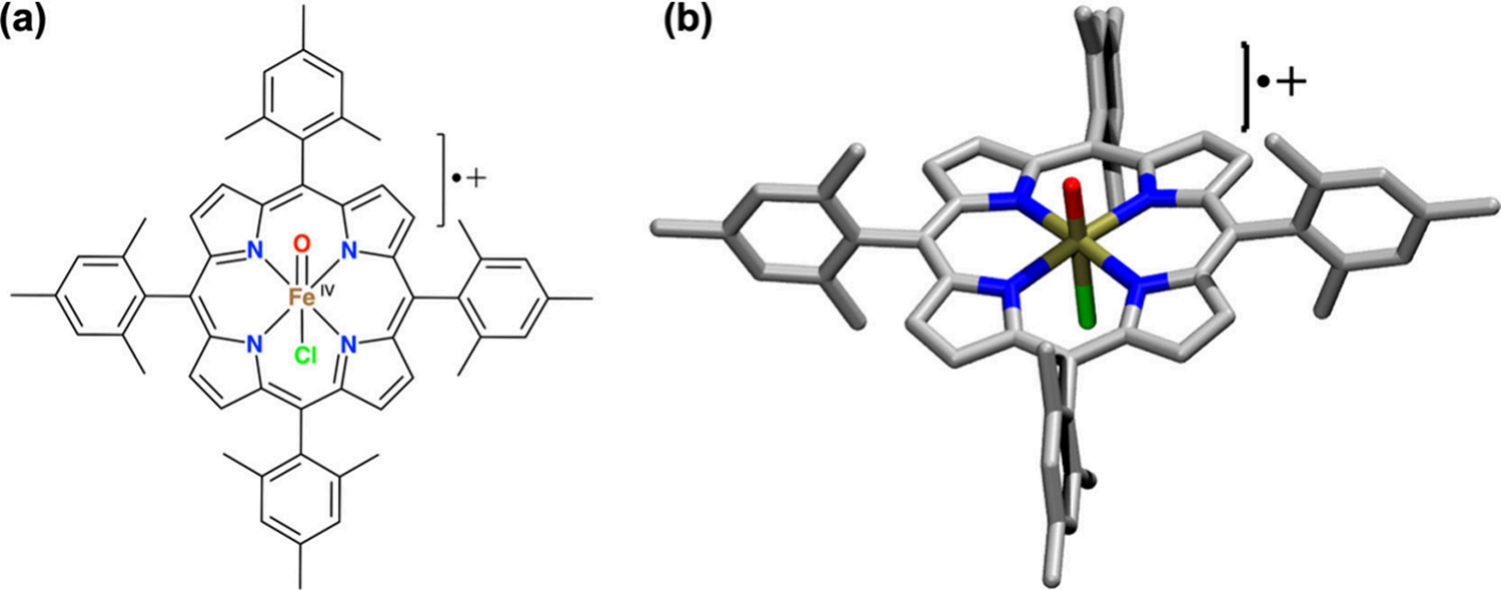
Synthetic Cpd I species, [Fe^IV^(O)­(TMP^•+^(Cl^–^))], where TMP is the shorthand notation for tetramesityl-porphyrin, is shown in (a) using the ChemDraw style structure, and in (b) using the 3D representation, similar to other oxo complexes as shown in Scheme 1b-c.

Thus, as shown in Scheme 2, the four mesityl ligands in the synthetic Cpd I species are donor groups, which occupy the meso positions of the porphyrin, and the axial ligand is chloride. In principle, these features preserve the basic characteristics of the natural Cpd I species. Nevertheless, it comes as a surprise to realize that, despite having analogous active species, there is a distinct difference between the KIE values obtained under enzymatic versus the nonenzymatic, in-solvent- laboratory conditions. As such, this raises the most natural question: *Why do P450 enzymes exhibit low KIEs despite all expectations to the contrary?* A likely rationale **is that the protein-fold of the enzyme makes the difference** between the low KIE of the P450 enzymes, and the very large KIE, due to QMT, **in the solution-phase reaction** of the synthetic Cpd I (Scheme 2).^28^

To address this counterintuitive mechanistic pattern, we have chosen two of the most popular bacterial P450s,^42,43^ P450_CAM_ and P450_BM3_, which represent different classes of P450 enzymes.^18,19^ To these, we added TauD, from the nonheme α-KG-dependent dioxygenase family.^20,21^ As we shall see, TauD utilizes a large tunneling during HAT, while the P450 Cpd I species harnesses the strong local electric fields (LEFs) of their protein-folds, which have larger barrier-lowering impact than QMT. Thus, our dichotomic HAT reactions lie at opposite ends of the KIE spectrum due to different LEF impacts from the respective enzyme-proteins folds. It is essential therefore, to elucidate the factors which operates in these two functionally analogous enzymes.

The above discussion leaves us with a major mechanistic puzzle: if the QMT effect is moderate in P450 enzymes, unlike in their synthetic oxo-iron counterparts^28^ (e.g., in Scheme 2), how do P450 enzymes maintain such highly efficient reactivity? What unique properties of the P450 enzyme overcompensate for the absence of the QMT effect in its reactivity? Accordingly, the present manuscript discusses the root cause of the observed low KIE values of P450 enzymes and investigates the ultimate contributor to their high reactivity, which compensates for the lack of QMT. In so doing, the manuscript comprehensively reconciles one of the longstanding mechanistic puzzles associated with enzymatic reactivity. As will be shown, the key factors are the significant LEFs of the enzyme protein-folds of P450 vis-á-vis the α-KG-dependent dioxygenases, which lack a significant LEF but use QMT to enhance the reaction rate, similarly to the synthetic Cpd I^28,40,41^ species (Scheme 2), which is devoid of protein-LEF effects.

## 2 Computational Methods

We used classical MD simulations to investigate conformational changes, while hybrid QM­(DFT)/MM calculations employed to explore reactivity, potential energy surface (PES), KIE, and other properties. In addition, we performed QM-only density functional theory (DFT) calculations for gas-phase studies. The details of all methods are systematically discussed below.

### 2.1 System Preparation and MD Simulations

The initial geometries for all cocrystallized enzymes were taken from the Protein Data Bank (PDB) with the following PDB IDs: 2CPP^44^ for P450_CAM_, 1JPZ^45^ for P450_BM3_, and 1OS7 ^46^ for TauD. Hydrogen atoms in the enzymes were added using the LEAP module of AMBER22,^47,48^ employing the ff14SB^49^ protein force field. Similarly, hydrogen atoms were added to the ligand molecules using the REDUCE tool in AMBER22.^47,48^ Next, we used the Antechamber module and Parmchk2 program, both built into AMBER22,^47,48^ to calculate the partial atomic charges and obtain the force field parameters, for all ligands, with both processes being facilitated by the built-in General Amber Force Field (GAFF).^50^ The GAFF force field employs the restrained electrostatic potential (RESP) method to calculate the partial atomic charges of the ligands using QM theory at the HF/6–31G* level.^51,52^ The force field parameters for the heme-porphyrin cofactor (Cpd I) of P450s were taken from previously published data.^53^ On the other hand, the force field parameters for the nonheme α-KG-dependent metal cofactor of TauD were generated using the built-in Metal Center Parameter Builder (MCPB)^54^ tool in AMBER22. We then solvated the enzymes/ligand systems in an octahedral box of TIP3P water,^55^ extending 10 Å from the enzyme surface. To maintain charge neutrality, Na^+^/Cl^–^ ions were added based on the enzymes’ inherent charges. Additionally, the PropKa tool^56^ was used to determine the protonation states of all titratable residues, which were carefully considered prior to the MD simulations. All MD simulations were performed using the GPU version of AMBER22,^47,48^ employing the ff14SB^49^ protein force field coupled with the TIP3P water model,^55^ as this combination has previously demonstrated excellent performance in both simulations and other QM/MM calculations.^57–59^

A general description of the various parameters and procedures used in MD simulations is provided in the SI (see Section S.1.).

### 2.2 A Brief Description of QM/MM Methods

Using a representative MD snapshot from the production MD ensemble, we proceeded with QM/MM geometry optimization. The details of the choice of the QM zones can be found in the SI (see Section S.2.). P450 calculations were performed in their two virtually degenerate ground states,^57^ the doublet and quartet, while the TauD calculations were carried out in its quintet ground-state.^60^ All calculations were performed using the ChemShell software package,^61,62^ where the QM zone was treated with TURBOMOLE^63^ and the MM zone with the DL_POLY program.^64^ The UB3LYP/def2-SVP (BS1) level of theory was applied for the QM zone, and the AMBER force field was used for the MM zone. Hydrogen link atoms were added to the dangling bonds at the boundary between the QM and MM zones with the charge-shift model.^61,62^ The protein and water molecules interact with the QM zone via electronic embedding,^65^ with their electrostatic effects included in the QM Hamiltonian through the incorporation of MM charges. The optimized reactant complex (RC) was subjected to a relaxed PES scan to locate the transition state (TS) geometry, and subsequently the product complex (PC). Both the TS and PC geometries were further reoptimized. Frequency calculations were performed for all geometries to confirm the nature of the ground state and TS, as well as to calculate the zero-point energy (ZPE) corrections. Finally, all optimized geometries underwent single-point energy calculations using the higher-level theory UB3LYP/def2-TZVP (BS2), with the energies being further refined using the Grimme D3 dispersion (GD3) correction. The choice of the level of theory is based on our previous experience working with enzyme mechanisms.^42,43,57,58,66^ All final reported energies are at the BS2 + GD3 + ZPE level of theory.

#### 2.2.1 KIE Calculations

All reported KIE values in this study were obtained from Eckart-based tunneling-corrected reaction rates.^67^ The detailed procedures and rationale behind the choice of this method can be found in the SI (see Section S.3.).

#### 2.2.2 Minimum Energy Pathway Calculations

All minimum energy pathway (MEP) calculations were performed using the Nudge Elastic Band (NEB) method^68^ incorporated in ChemShell.^61,62^ Unlike the mass-weighted displacement used in intrinsic reaction coordinate (IRC) calculations, the NEB method maps the MEP, using Cartesian displacements in angstroms (Å). The detailed procedure for the NEB method can be found in the SI (see Section S.4.).

### 2.3 QM-Only DFT Calculations

While performing the gas-phase QM-only DFT calculations, we used the QM coordinates derived from the QM/MM-optimized geometry. The Gaussian 16 Revision B.01 software package^69^ was used to perform all frequency calculations required to determine the gas-phase reaction rate and, consequently, the KIE. The tunneling-corrected Eckart-based rate of the reaction in the gas phase was calculated using the TheRate program.^70^ We further verified that upon reoptimization of those QM coordinates in the gas phase using UB3LYP/def2-SVP level of theory, also yields large KIE values. To be consistent with the Turbomole QM/MM calculations we used the VWN­(V) exchange correlation functional for B3LYP calculations in Gaussian 16 package. Additionally, the ORCA 6.0 software package^71^ was employed to calculate the MEP using the NEB method.^68^ For all QM calculations, we maintained consistency by using the same level of theory as in the QM/MM calculations.

### 2.4 Local Electric Fields and Interaction Energy Calculations

The impacts of local electric fields (LEFs) induced by enzyme folds on the catalytic site were calculated using the TITAN software program.^72^ Since the catalytic sites of P450s and TauD enzymes are the respective iron-oxo moieties (see Scheme 1b,c), we chose the Fe=O bond as the vector for calculating the LEFs of the enzyme.^73–75^ For the LEF calculations, we used the point charges of the MM atoms generated by ChemShell during the geometry optimization, and for the QM atoms, we considered the QM/MM derived natural charges obtained from the natural population analysis.^76^ Next, we computed the interaction energy (Δ*E*) resulting from the interaction between the LEFs (*F⃗*_*x*_) and the dipole moment (*μ⃗*_*x*_) of the QM/MM optimized geometry using the eq 2, where θ serves as the angle between two vectors.^77,78^ The exact value of θ was calculated using the coordinates of the *F⃗*_*x*_ and the components of *μ⃗*_*x*_. Detailed mathematical derivations are provided in the SI (see Section S.5).

2 $$\Delta E=4.8{\mathrm{F⃗}}_{x}•{\mathrm{\mu ⃗}}_{x}\;\left[=\left|{\mathrm{F⃗}}_{x}\right|\left|\mathrm{\mu ⃗}\right|\cos\left(\theta \right)\right]$$

Here, the units of Δ*E*, *F⃗*_*x*_ and *μ⃗*_*x*_ are in kcal/mol, V/Å and Debye, respectively.

## 3 Results and Discussion

### 3.1 Modeling the Active Oxidant and Reactivity Calculations

In their crystal structures, the active sites of all three chosen enzymes, P450_CAM_, P450_BM3_, and TauD, are in their respective resting state. Therefore, we first modeled these species in their active oxidant states; the respective iron-oxo species (doublet and quartet states for P450s, and a quintet state for TauD). Subsequently, we performed MD simulations to capture all the conformational changes that may occur in the respective active states. Thereafter, we selected a representative MD snapshot and subjected it to QM/MM geometry optimization and PES scan to obtain a comparative reactivity pattern under common conditions. In all cases, we selected the C–H having the correct stereochemistry and highest hydrogen abstraction reactivity. See barriers and geometries in Figure 1.

**Figure 1:**
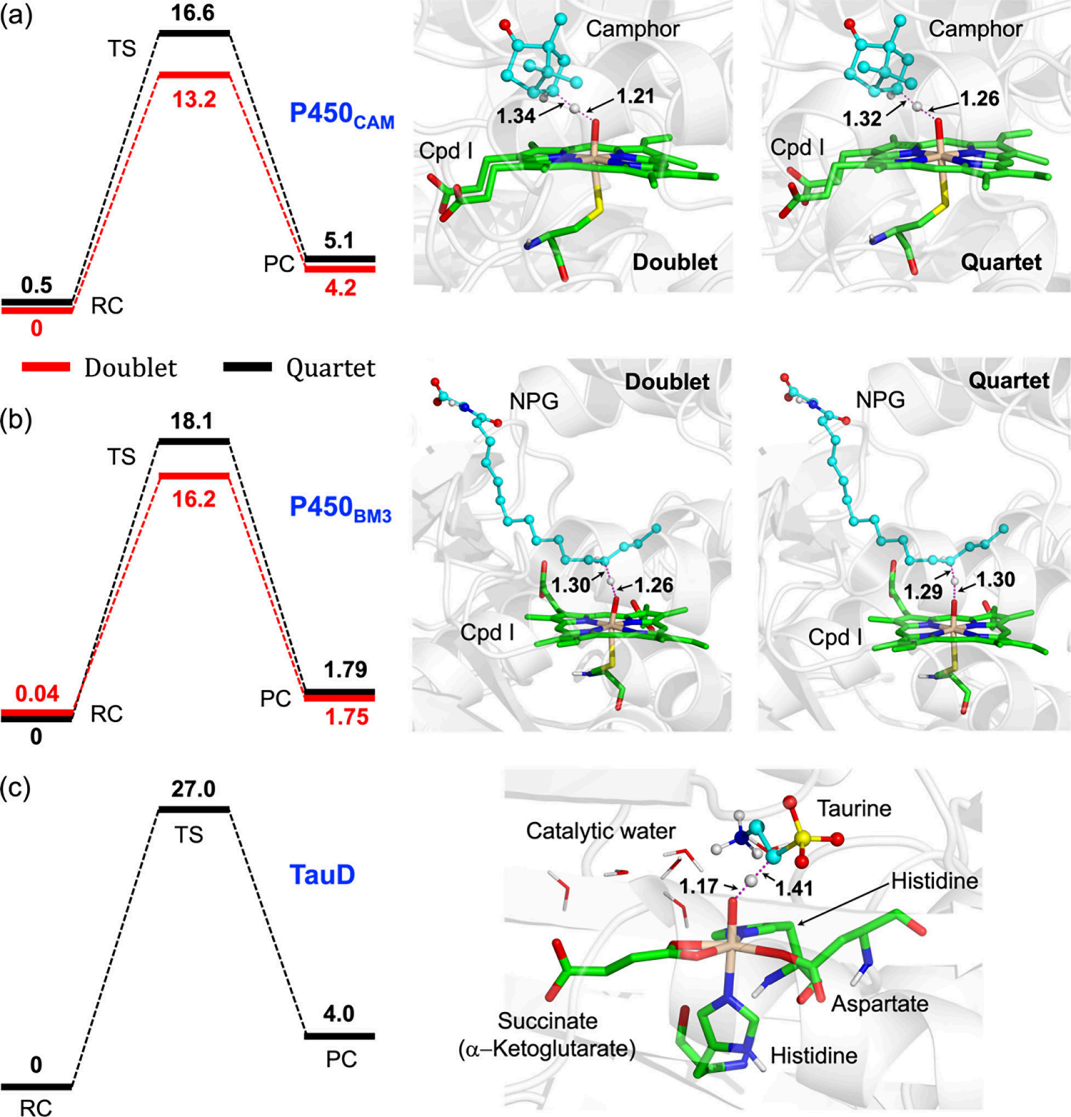
QM/MM results for the HAT reactions. Activation energies (in kcal/mol) and reaction profile diagrams (left) for the HAT reaction, and the corresponding QM/MM-optimized TS geometries for (a) P450_CAM_, (b) P450_BM3_, and (c) TauD enzymes. The red and black lines in the energy profile diagrams, in (a) and (b), represent the doublet and quartet energy profiles, respectively. Energies were calculated using the BS2 + GD3 + ZPE level of theory. All energies are given relative to the RC in kcal/mol. Distances are given in Å. Note that, in all cases, the product state following the HAT event is labeled as the PC. The NPG refers to the substrate, N-palmitoylglycine. The optimized geometries for the RC and PC are collected in Figure S2 in the SI.

As shown in Figure 1a-b, the energy barriers for HAT in both the doublet and quartet spin states of P450_CAM_ and P450_BM3_ are moderate, typically ranging from 13.2 to 18.1 kcal/mol, and resulting in endothermic product complexes (PC species). This observation is in good agreement with previously published results.^57^ Similarly, as shown in Figure 1c, the barrier required for the HAT in the TauD enzyme is approximately 27 kcal/mol, which is ∼10–12 kcal/mol higher than the barriers for P450s (see Figure 1a-b) **under identical conditions**. At this point, one may be curious about the relatively higher barrier observed for the TauD enzyme. After careful investigation, we found that its origin is primarily geometric and structural in nature (see Section S.6., Table S1 and Figure S1 in SI). Thus, using these barriers, one can calculate the respective reaction rate (*k*) from the Eyring equation^2,79^ (in eq 3), derived from TST. However, this approach may not reflect the true reactivity, as it does not account for QMT effects. As such, depending on the extent of QMT, the actual reactivity of a given reaction may be greater than the calculated one with eq 3:

3 $$k=\left(\frac{k_{B}T}{h}\right)e^{-\Delta G^{‡}/RT}$$

Here, *k*_B_ is the Boltzmann constant, *h* is Planck’s constant, *R* is the universal gas constant, *T* is the temperature, and Δ*G* ^‡^ is the Gibbs energy of activation. Since the determination of precise QMT effects in a chemical reaction remains challenging, the most common approach is to apply a QMT correction to the reaction rate,^1^ after which the QMT-corrected KIE values can be evaluated and compared with the experimental results. This is done in section 3.2.

### 3.2 Exploring the Effect of QMT on Reactivity

To determine the effect of QMT on HAT reactivity, we first calculated the QMT-corrected rate, as discussed in section 2.2.1 (and S.3. in the SI), and then evaluated the KIE for the respective reaction. Table 1 presents the calculated KIE values alongside the experimentally determined ones. Note that the QM/MM-derived KIE values correspond to those experimentally measured in an enzymatic environment, while the QM-derived KIEs are obtained by considering only the QM zone of the QM/MM setup, i.e., in the absence of enzyme folds and catalytic water.

**Table 1:** QM/MM-Calculated (in Enzymatic Environment) and QM-Only-Calculated (in Nonenzymatic Environment) KIE Values as Well as Experimentally Determined Ones, for P450_CAM_, P450_BM3_, and TauD^a^

	KIE (P450_CAM_)		KIE (P450_BM3_)		KIE (TauD)
	QM/MM	QM-only	QM/MM	QM-only	QM/MM
Doublet	5	35.7^c^	6	68.0^c^	NA
Quartet	5.9	81.2^c^	6.2	57.1^c^	
Quintet	NA				62.3^b^
Experimental	1.23 ± 0.05		7.3 ± 2		58

a For P450_CAM_ and TauD, the experimentally determined KIE values are for the respective natural substrates, camphor and taurine, respectively. The KIE for P450_BM3_ is for *p*-xylene as the substrate. To our knowledge, the KIE for P450_BM3_ with its natural N-palmitoylglycine (NPG) substrate has not yet been experimentally determined (see Table S2 for Eyring-only KIE values).

b Note that the zwitterionic form of taurine in TauD for the gas phase QM-only calculations could not be stabilized.

c The KIE values obtained from the QM-only UB3LYP/def2-SVP reoptimized coordinates are 29.4 (doublet) and 42.3 (quartet) for P450_CAM_ and 30.5 (doublet) and 50.2 (quartet) for P450_BM3_.

Table 1 shows that, in all cases, the QM/MM-derived KIE values generally match the experimentally determined ones.^27,38,39^ For example, the KIE for P450_CAM_ and P450_BM3_ in QM/MM calculations for both spin states are small, and so are the experimental values.^38,39^ This agreement demonstrates the absence of QMT effects in the P450 catalyzed HAT reactions, thereby supporting the reactivity predictions by the ΔG^‡^ quantity and eq 3. In addition, the difference between the KIEs obtained in the respective doublet and quartet spin states is also small, indicating the almost degenerate nature of these states in the active oxidant Cpd I (in its ground-state geometry). Note that **for the QM-only P450s, the KIE values are high, in contrast to the low KIEs for the in-protein (QM/MM) reactions**. This issue is further elaborated below.

By contrast to the small KIE­(P450) values, a large QM/MM-calculated KIE (62.3) is computed (Table 1) for the TauD enzyme, in a good match with the experimentally obtained^27^ KIE value (58). This large calculated KIE for TauD is an indicator of QMT, which augments the corresponding reactivity relative to the one predicted from the energy barrier in Figure 1c. As such, it is tunneling that enables TauD to efficiently perform the hydroxylation reaction by cutting through the barrier.

#### 3.2.1 Prediction of KIE at Low Temperatures

We further calculated the QM/MM-derived KIE values at several temperatures below room temperature, down to 200 K. As shown in Figure 2, the KIE increases sharply below 250–260 K for both P450s, highlighting the dominant role of QMT in their reactivities at lower temperatures. Additionally, we see that as temperature decreases, the gap between the doublet and quartet states KIEs increases in both P450s, indicating the lifting of degeneracy in their reactive spin states at lower temperatures.

**Figure 2:**
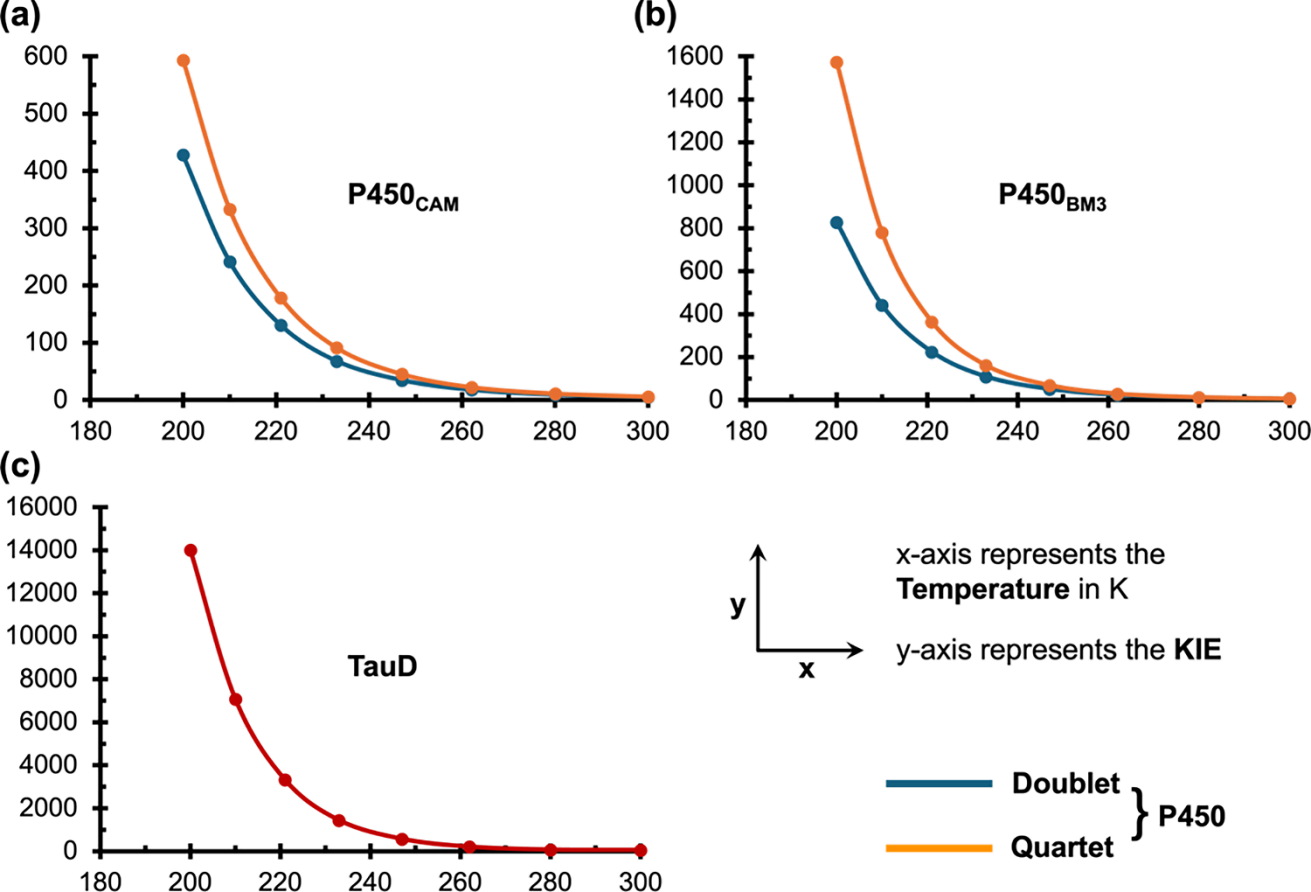
Predicted KIE values (QM/MM levels) as a function of temperature for HAT reactions catalyzed by (a) P450_CAM_, (b) P450_BM3_, and (c) TauD enzymes.

Similarly, the TauD reaction reveals also an increase in KIE with decreasing temperature. However, the rate of KIE increase is significantly higher compared to the P450 cases. For instance, the TauD enzyme exhibits an exceptionally high KIE of 14,000 at 200 K, by comparison to approximately 600 for P450_CAM_ and 1,600 for P450_BM3_ (see Figure 2). Thus, compared to TauD, the P450 reactions exhibit much lower KIE values at lower temperatures. This further indicates that the mechanistic puzzle we are investigating is not confined to room temperature; it persists at lower temperatures.

Nevertheless, the above trends lead to the following important question: *Why does QMT become so prominent, resulting in higher KIEs with decreasing temperatures?* Although there is no simple relationship between QMT and temperature, it can be deduced that at low temperatures, thermal activation may be insufficient to overcome the reaction barrier. In such cases, the lighter hydrogen isotope can show significant reactivity via QMT, while the heavier deuterium isotope becomes less effective, thus giving rise to significantly higher KIE values at low temperatures.

#### 3.2.2 Gauging the Role of Enzyme Protein-Folds. QM/MM vs QM-Only Studies

The resulting low KIEs in both calculated and experimentally derived values for P450-catalyzed HAT reactions suggests that in P450s, there is a key factor which reduces the effect of QMT, and leads thereby to low KIEs. To investigate this issue further, we evaluated the KIE of each P450 reaction in the absence of the protein folds, considering only the reactive molecular components. Indeed, as shown in the QM-only data in Table 1, in the absence of protein folds the KIE for P450-catalyzed reactions is now several times larger than the value obtained in their presence. This is consistent with other HAT reactions discussed earlier. In addition, this observation closely parallels the high experimental and computational KIEs observed for HAT reactions catalyzed by synthetic Cpd I species (e.g., in Scheme 2).^28,40,41^ It follows therefore that the enzymatic protein-folds in P450s have a unique impact that diminishes both the barrier and QMT effect on their HAT reactivity and leads to low KIE values.

Nevertheless, this still leaves an important question: How do the enzyme folds reduce the effect of QMT and, consequently also the KIEs? This is discussed in the following section.

### 3.3 Origin of the Low KIE in P450s

Low KIEs are typically observed due to the near absence of QMT in chemical reactions, and QMT depends on the probability of an atom penetrating through the barrier during the reaction. Therefore, if the penetrating atom is the same in all reactions (e.g., HAT), the shape of the PES becomes the key factor that governs the effect of QMT on reactivity. Accordingly, a narrower PES width increases the probability of QMT, while a broadened PES lowers QMT.^1,80,81^

It has been previously demonstrated that the imaginary frequency of the TS is a reliable measure of the PES shape/width, with larger magnitudes corresponding to narrower PES and vice versa.^80,81^ Indeed, as seen in Table 2, the imaginary vibrational frequencies of the QM/MM-derived TS are smaller than those of their QM counterparts for both P450_CAM_ and P450_BM3_, indicating a broader PES in the presence of the enzyme folds, which diminishes QMT and consequently leads to the observed lower KIEs.

**Table 2:** Imaginary Vibrational Frequencies (in cm^–1^) of TSs Obtained from QM/MM Calculations (with Enzyme Folds and Catalytic Water) and QM-Only Calculations (without Enzyme Folds and Catalytic Water)

Imaginary vibrational frequencies of TS geometries (in cm^–1^)					
	P450_CAM_		P450_BM3_		TauD
	QM/MM	QM-only	QM/MM	QM-only	QM/MM
Doublet	1341.1i	1544.0i	1451.8i	1918.0i	NA
Quartet	1418.8i	1908.5i	1207.3i	1830.1i	
Quintet	NA				1723.1i

Similarly, the imaginary vibrational frequency obtained for the TauD-catalyzed HAT reaction is 1723.1i cm^–1^, which is higher than those for both P450 enzymes. This further supports the presence of a narrower PES in TauD compared to the PES in P450 reactions, as the root cause of stronger QMT effects and consequently higher KIE values during TauD reactivity.

#### 3.3.1 Mapping the Shape of the PES

The previous section establishes how the shape of the PES plays a crucial role in determining the extent of QMT and the resulting KIEs. To reach a definitive conclusion, we mapped the exact shape of the PES by performing minimum energy path (MEP) calculations, as displayed in Figure 3 (see Section 2.2.2 and S.4., in the SI, for detailed methods).

**Figure 3:**
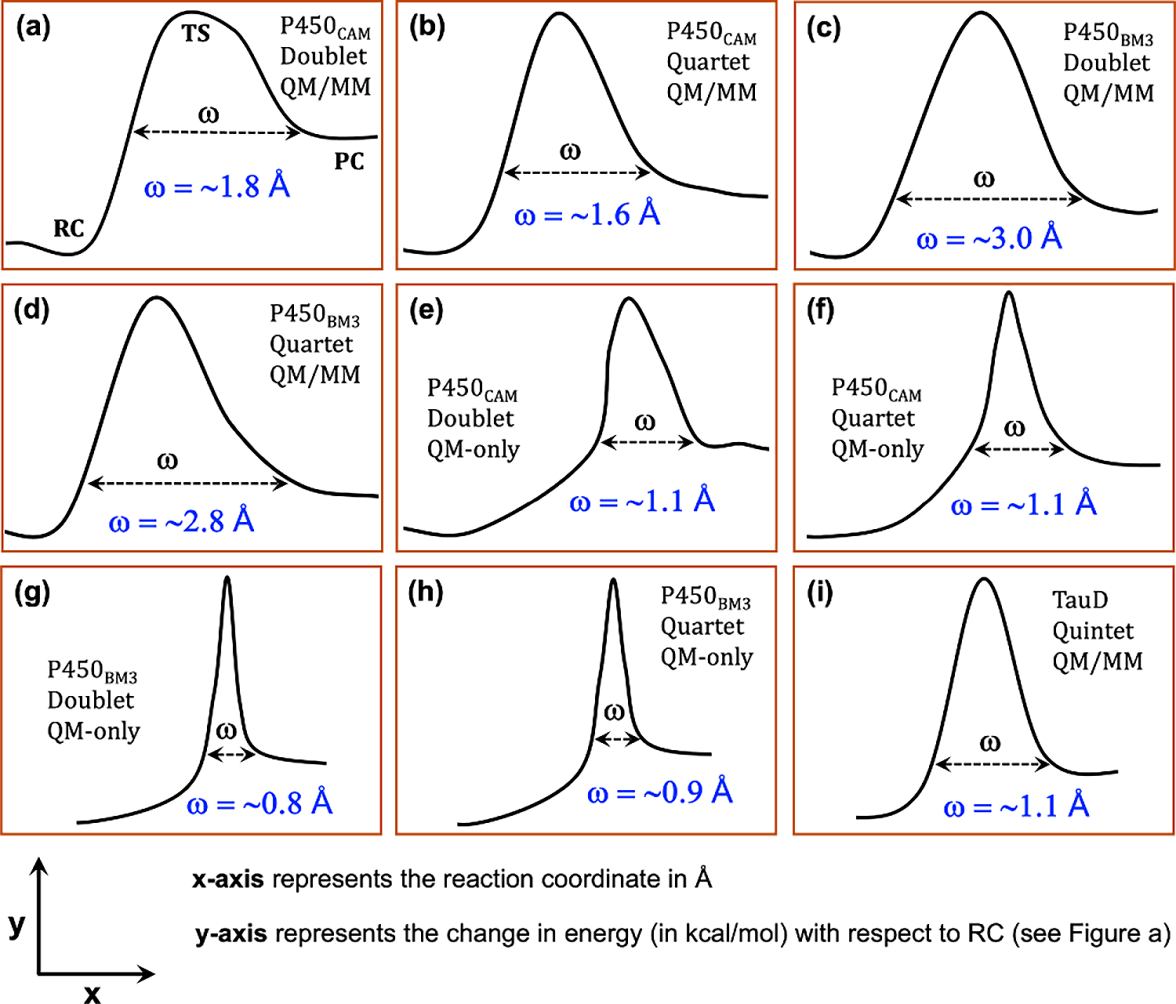
Shapes and barrier widths (ω) of the potential-energy surface (PES) derived from MEP calculations of the H-abstraction reactions at the BS1 level of theory, for P450_CAM_, P450_BM3_ and TauD. Parts (a) and (b) show the ω values which are calculated for the QM/MM barriers of the doublet and quartet state reactions of P450_CAM_, while part (c) and (d) show the corresponding ω values for the QM/MM barrier of P450_BM3_. Parts (e)-(h) provide the ω values for the QM-only barriers at the doublet and quartet spin states for both P450_CAM_ and P450_BM3_. Similarly, part (i) shows the ω value for the QM/MM barrier of TauD in the quintet state. Refer to Figure 1 for the energetics.

As such, in the case of P450_CAM_, the barrier width in the QM/MM calculations is approximately 1.8 Å for the doublet state (see Figure 3a) and 1.6 Å for the quartet state (see Figure 3b). By contrast, for the QM-only reactions, **the barrier-width is only 1.1 Å for both states** (see Figures 3e and 3f). A similar trend is also observed in P450_BM3_, where the barrier width in the QM/MM ranges from 2.8 to 3.0 Å (see Figures 3c and 3d), whereas in the QM-only case, it is less than 1.0 Å (see Figures 3g and 3h). In all cases, the barrier width, ω, is calculated at the energy level of the product exit (PC). This point is determined as the stage where the energy no longer decreases significantly with the progress of the reaction coordinate in the MEP and becomes essentially flat. Thus, the wider barrier obtained in QM/MM calculations, compared to QM-only calculations, along with the visual comparison of the PES, clearly *reveals that the PES in the presence of the enzyme folds (QM/MM) is significantly broader than in their absence in the QM-only processes*. This is further supported by the QM/MM-derived reduced barrier widths (1.1 Å, see Figure 3i), which corresponds to the large KIE value observed in the TauD-catalyzed HAT reaction.

Thus, ***these findings strongly suggest that the unique protein-enzyme folds of P450s are the primary root-causes of the PES broadening***, *which is the ultimate cause of the diminished QMT effect, thereby resulting in the low KIEs values observed in their HAT reactions*. Indeed, the synthetic P450-like Cpd I species in Scheme 2, leads (in solution) to a very large KIE value, with substantial QMT, due to a narrow PES.^28^ By contrast, the in-enzyme Cpd I species do not lead to QMT.

At this point, one may wonder whether the narrowed barrier width observed (see Figures 3e-i) is sufficiently narrow to facilitate hydrogen atom tunneling. In general, if the width of the PES is comparable to the wavelength of the associated atomic particle (hydrogen, in our case), the likelihood of QMT increases.^28^ Since determining the exact wavelength of the transferring hydrogen atom in a particular reaction is challenging,^82–84^ we estimated the wavelength of a free-hydrogen-atom as ∼1.45 Å, at ordinary temperatures using the equipartition theorem and the de Broglie relation (see Section S.7 in the SI for detailed mathematics). This value serves as a useful benchmark. As such, the barrier widths obtained in our narrowed PESs (see Figures 3e-i) are fairly comparable to the wavelength of a free hydrogen atom, supporting the presence of QMT. The somewhat smaller widths in Figure 3e-i may simply reflect the fact that the bonded-H atoms are partially positively charged and hence smaller than a free-H atom.

### 3.4 The Strategic Role of Local Electric Fields in Enzyme Catalysis

So far, it has been observed that P450 enzymes do not rely on QMT but instead maintain high reactivity by lowering reaction barriers. Furthermore, previous studies have shown that most P450 enzymes exhibit moderate H-abstraction barriers (not exceeding 20 kcal/mol).^57,58,66^ By contrast, TauD exhibits relatively higher reaction barriers compared to those of the P450 enzymes under identical conditions, and its reactivity is enhanced through QMT. This raises therefore an intriguing question: How does nature accommodate these two distinct mechanistic strategies to enhance reactivity in HAT reactions for two functionally analogous enzymes?

To address this puzzle, we investigated the effects of local electric fields (LEFs) induced by enzyme folds on the catalytic site (Figures 4a-c). Following Warshel’s theoretical conceptualization^3–5^ of the strategic role of LEFs in enzyme catalysis, and the experimental foundation laid by the Boxer group,^6,7^ several recent studies^73–75,85^ have emerged that continue to explore and expand upon this idea. For instance, Matta and co-workers studied how a strong external electric field influences the kinetics of double proton transfer in the formic acid dimer.^86^

**Figure 4:**
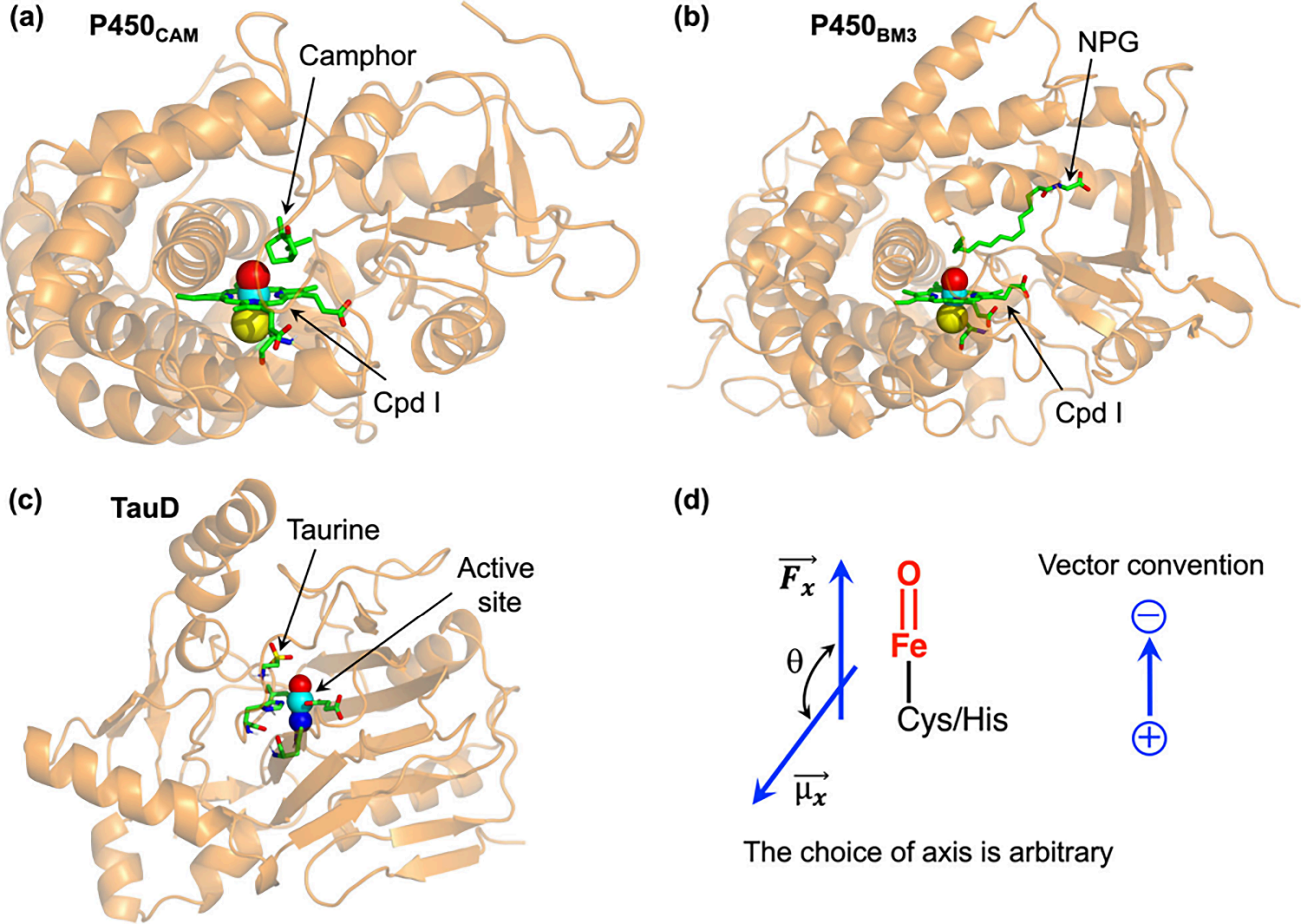
Representations of enzyme-protein-folds, composed of various secondary structures such as α-helices, β-sheets, and loops, for (a) P450_CAM_, (b) P450_BM3_, and (c) TauD. The active sites are shown by use of stick and vdW representations. Here, NPG refers to the substrate, N-palmitoylglycine. (d) A schematic representation of the iron-oxo catalytic site and the interaction between the dipole moment (*μ⃗*_*x*_) and LEF (*F⃗*_*x*_) vectors, where θ denotes the angle between the two vectors.

In the current study, we quantified the LEFs generated by the enzyme folds for the three enzymes (Figures 4a–c) at their respective catalytic site (Figure 4d) for both the reactant (RC) and transition state (TS) geometries. Thereafter, based on the dipole moment, we evaluated the interaction energy of each reactive state (RC and TS), induced by these electric fields, using eq 2. The results are summarized in Table 3.

**Table 3:** Data for the LEFs (*F⃗*_*x*_), Dipole Moment (*μ⃗*_*x*_) Vectors, and Interaction Energies (Δ*E*) from eq 2, for Three Enzymes

Enzymes	Multiplicity	States	LEF (V/Å)	Dipole Moment (Debye)	θ (Degrees)	Δ*E* (kcal/mol)	ΔΔ*E* (kcal/mol) (Δ*E*_TS_ – Δ*E*_RC)_
**P450**_**CAM**_	Doublet	RC	0.011493	6296.99	167.49	–339.15	**–3.90**
		TS	0.011588	6291.86	168.57	–343.05	
	Quartet	RC	0.011479	6297.08	167.08	–338.18	**–2.74**
		TS	0.011609	6294.06	166.42	–340.92	
**P450**_**BM3**_	Doublet	RC	0.001887	4976.06	101.68	–9.12	**–23.17**
		TS	0.003685	4973.77	111.54	–32.29	
	Quartet	RC	0.001894	4976.02	101.81	–9.26	**–8.61**
		TS	0.003528	4973.46	102.24	–17.86	
**TauD**	Quintet	RC	0.002305	2131.34	111.0	–8.45	**4.20**
		TS	0.001197	2131.95	110.31	–4.25	

As shown in Table 3, the interaction energies (Δ*E*) for both RC and TS states across all three enzymes are negative, indicating that LEFs stabilize these states. Simultaneously, the relative stabilization energy (ΔΔ*E*) of the TS vis-à-vis the RC reflects opposite patterns in P450s vs TauD:

(i) In P450s, the TS is consistently more stabilized by the LEF than the corresponding RC, and hence, the interaction with the LEF lowers the reaction barrier and broadens it. Thus, the LEFs of P450s reduce reaction barriers, and bypasses the tunneling option.
(ii) By contrast, in TauD, the ΔΔ*E* value is positive because the LEF stabilizes the RC more than the TS. Consequently, **the TauD protein increases the reaction barriers**, while endowing the reactions with significant tunneling.
(iii) An additional aspect of this analysis is that the ΔΔ*E* values for the doublet states of the two P450s are more stabilizing than those for the corresponding quartet states. This qualitatively aligns with the lower doublet-state barriers compared to the quartet counterparts (see Figure 1).

In summary, therefore, the LEF in TauD disfavors the formation of the TS relatively to the reactant state (RC), thereby TauD causes the reaction to proceed by tunneling. By contrast, the LEFs of P450s lower the TS energy and thereby broaden the barrier, thus enhancing the reaction rate at the expense of losing the tunneling effect.

## 4 Conclusion

This study explores the origins of the low vs high KIEs observed in P450 vs TauD enzymes. We find that the LEF of P450 proteins broadens the energy profiles, for the H-abstraction reactions, and thereby diminishes the influence of quantum mechanical tunneling (QMT), giving rise to low KIEs. By contrast, the TauD protein narrows the energy profile, and thereby the enzyme utilizes QMT to enhance reactivity and gives rise to high KIE values. Our study further demonstrates that the room-temperature KIE differences between P450s and TauD are consistent also at lower temperatures.

As such, our findings highlight also the importance of enzyme protein-folds in determining the reaction mechanism. For P450s, the protein-folds create, at the catalytic site, significant local electric fields (LEFs), which reduce the barriers and broaden the potential-energy profiles, thus compensating for the diminished QMT effect due to the protein. Hence P450s maintain high reactivity. By contrast, the LEF imposed by the protein-folds of TauD interacts unfavorably with the H-abstraction TS, thus increasing and narrowing the reaction barrier. As such, the TauD reactivity is compensated due to QMT.

This comparison of the two enzyme-types highlights the manners whereby nature accommodates two distinct mechanistic strategies to support the HAT reactivity, due the specific protein folds of the respective enzymes. This outcome accounts for the crucial role of the strategic positioning of protein-folds in enzyme catalysis.

In conclusion, our study connects the role of local electric fields (LEFs) of proteins to the observed disparity in KIEs and relative reactivities (reaction barriers), in two families of enzymes. In so-doing the study resolves a longstanding mechanistic enigma that clarifies the unique features of two enzymatic families which are very similar and yet also very different. In fact, the LEF effect in P450 and the QMT observed in TauD reflect the evolutionary diversity of enzymes that perform hydrogen abstraction reactions.

Is Nature a good strategist? Well, in the sense that variety is useful, *Nature seems to create a necessary variety in tweaking the reactivity properties of enzymes, by letting them utilize/select two different reactivity-effects*.

## Supplementary Material



## Notes

Information on the methodologies, the calculation of the hydrogen atom’s wavelength, detailed mathematics for finding the angle between two vectors, figures of the QM/MM-optimized snapshots, and the coordinates of the QM zone from various QM/MM-optimized geometries (PDF)

## #

S.K.: Department of Chemical Sciences, Tezpur University, Napaam, Tezpur, Assam-784028, India
