# Supplementary material for: Local Electric Fields Originate Unusual Kinetic Isotope Effects in the Hydrogen Abstraction Reactions of the Functionally Analogous P450 and TauD Enzymes
Source: J Am Chem Soc. 2025 Sep 17;147(39):35576–86. doi: 10.1021/jacs.5c10488 (PMC12498400; doi:10.1021/jacs.5c10488)
Supplement: Supplementary file 1 [file ja5c10488_si_001.pdf]

Local Electric Fields Originate Unusual Kinetic Isotope Effects in the Hydrogen Abstraction Reactions of the Functionally Analogous P450 and TauD Enzymes

Surajit Kalita, David Danovich, and Sason Shaik*

Institute of Chemistry, The Hebrew University of Jerusalem, Edmond J. Safra Campus, Givat Ram, Jerusalem 9190401, Israel.

Table of Contents

S.1. General Description of MD Simulations **S2**

S.2. Choice of QM Zones **S2**

S.3. KIE Calculations **S3**

S.4. Nudge Elastic Band Method **S3**

S.5. Finding the Angle Between the Dipole Moment and Local Electric Field (F=O) Vector **S4**

S.6. Understanding the Origin of the Reaction Barrier in TauD Enzyme **S5**

S.7. Calculation of de Broglie Wavelength of a Hydrogen Atom at Temperature 300 K **S6**

Figure S1: Transition state structure of the TauD enzyme without the catalytic water molecules included in the QM zone. All distances are given in Å. **S6**

Figure S2: Ball-and-stick representation of the QM zone in the QM/MM-optimized reactant and product complexes (RC and PC) of all three enzymes. The enzyme folds are shown as transparent white cartoons. Here, Cpd I and NPG refer to Compound I and N-Palmitoylglycine, respectively **S8**

Table S1: Spin densities of the Fe and O atoms in the iron-oxo moiety, and the substrate carbon atom involved in H-abstraction, for all three reactive species. **S5**

Table S2: Eyring-based KIE values obtained from QM/MM calculations in the enzyme environment. **S8**

References **S9–S11**

QM coordinates of the QM/MM optimized species **S11–S27**

S.1. General Description of MD Simulations

After completing the system setup, we performed a two-step minimization process on the entire enzyme-ligand system to avoid unwanted contacts and steric clashes that may arise during system preparation. In the first step, only the water molecules were minimized, while in the second step, the entire system was minimized. For both steps, we first applied 5000 steps of the steepest descent algorithm, followed by 5000 steps of the conjugate gradient algorithm. Subsequently, the system was gradually heated from absolute zero to 300 K in the NVT ensemble over 50 ps. After reaching the target temperature, the system was maintained at 300 K for 1 ns using the Langevin thermostat[1] in the NPT ensemble. During this phase, we used the Berendsen barostat[2] with a target pressure of 1 atm, a collision frequency of 2 ps, and a pressure relaxation time of 1 ps. Finally, the system was equilibrated for 3 ns under the same protocol.

After equilibration, the system underwent a productive MD run for 100 ns using a multitrajectory approach, where the MD run was restarted after 50 ns with a random velocity. Additionally, we performed replica simulations to ensure the consistency of the obtained results. During the production MD simulation, the Monte Carlo barostat[3] was employed, as it provides better thermodynamic accuracy and maintains consistency in the NPT ensemble. The SHAKE algorithm[4] was applied to constrain the vibrations of fast-moving hydrogens. Long-range electrostatic interactions were treated using the Particle Mesh Ewald (PME) method[5], while van der Waals forces were calculated with a cutoff of 12 Å using the Lennard-Jones potential method. All MD simulations were performed using the GPU version of AMBER22[6,7], and the inbuilt CPPTRAJ module was used to analyze the results. The VMD[8] and PYMOL[9] software packages were used to visualize the trajectories and create figures.

S.2. Choice of QM Zones

Before starting the QM/MM calculations, it is essential to determine the QM zone. For P450s, we used the standard QM zone[10], which includes a truncated porphyrin ring, the axial cysteine residue as HS^-, and the substrate molecule. In the case of $P450_{CAM}$, the entire camphor molecule was included in the QM zone, while for $P450_{BM3}$, only the most relevant portion of the N-Palmitoylglycine (NPG) was kept in the QM zone, based on previous studies.[11] Similarly, for the TauD enzyme, we included all residues in the QM zone that bind to the iron-oxo complex: two

histidine residues, one aspartate, succinate (α–ketoglutarate), and the substrate molecule, taurine. Similar to previous computational studies, we used succinate as the bidentate ligand.[12-14] Additionally, we included six water molecules in the QM zone, which were found to maintain a consistent water channel in the active site throughout MD simulations. These water molecules also interact strongly with the positively charged N-group of the taurine substrate, helping to stabilize its zwitterionic configuration.

S.3. KIE Calculations

In order to calculate the KIE, the reaction rate must first be determined. Accordingly, we used the ChemShell-built harmonic transition state theory (HTST) to calculate the reaction rate, which employs the energy and Hessian at the RC and TS geometries.[15,16] To begin, we calculated the Hessians for both RC and TS geometries using the keyword 'thermal = true' in the DL-FIND module of ChemShell. The rate was then determined by performing a separate DL-FIND calculation with the keyword 'rate = true.' Once the rate for the lighter isotope was calculated, we changed the mass of the targeted atom to its heavier isotope using the keyword 'mass.' The overall Hessian was reweighted, and the rate was recalculated, followed by the determination of the KIE.

The rate, and thus the KIE, obtained in our study is a tunneling-corrected rate, calculated using an Eckart-based method.[17] This method uses an analytical potential energy function along the adiabatic minimum energy path (MEP), or intrinsic reaction coordinate (IRC) in mass weighted coordinates, to account for tunneling effects. Other groups[18-20], including our own[21-23], have previously demonstrated the reliability of the Eckart-based method in calculating tunneling-corrected KIE values. These studies have shown that the one-dimensional Eckart-based KIE values closely match those from multidimensional[24] calculations as well as experimental[24] KIE values in several reactions. Therefore, we chose the Eckart-based method for our calculations due to its simplicity, reliability, and computational efficiency, particularly for our large QM/MM systems. As a result, all the reported KIE values in this manuscript are tunneling-corrected Eckart-based KIE values.

S.4. Nudge Elastic Band Method

We performed nudge elastic band[25] (NEB) calculations to derive the exact shape of the one-dimensional PES. The NEB method connects the reactant and product complexes by finding the

MEP. Using the DL-FIND module of ChemShell,[15,16] we enabled the NEB calculations with the keyword 'neb = frozen,' which freezes the end points of the NEB path. The optimized RC and PC geometries were used as the end points. For selecting the number of images, we chose 8, as recommended by ChemShell. We employed BS1 level of theory for all NEB calculations.

S.5. Finding the Angle Between the Dipole Moment and Local Electric Field (F=O) Vector

Let the coordinates of two atoms (e.g., Fe and O in our study) be:

$$A_1 = (a_1, a_2, a_3) \qquad\qquad A_2 = (b_1, b_2, b_3)$$

The Local Electric Field (LEF) vector, denoted by $\vec{F}_x$, defined along the bond connecting the two atoms, is given by:

$$\vec{F}_x = A_2 - A_1$$

Note: The choice of axes is arbitrary

The dipole moment vector $\vec{\mu}_x$ of the optimized enzyme system is represented as:

$$\vec{\mu}_x = \vec{\mu}_1\hat{\imath} + \vec{\mu}_2\hat{\jmath} + \vec{\mu}_3\hat{k}$$

Since $\vec{F}_x$ does not necessarily pass through the origin of the enzyme's coordinate frame, one could, in principle, shift $\vec{F}_x$ to intersect $\vec{\mu}_x$ at the origin by computing the shortest distance *d*, given by:

$$d = \frac{\sqrt{\{a_2(b_3-a_3)-a_3(b_2-a_2)\}^2+\{a_1(b_3-a_3)-a_3(b_1-a_1)\}^2+\{a_1(b_2-a_2)-a_2(b_1-a_1)\}^2}}{\sqrt{(b_1-a_1)^2+(b_2-a_2)^2+(b_3-a_3)^2}}$$

However, this shifting is not necessary, as the angle between two vectors is invariant under translation. Thus, we can directly compute the angle θ between $\vec{F}_x$ and $\vec{\mu}_x$ using the dot product formula:

$$\theta = \cos^{-1}\left(\frac{\vec{F}_x \cdot \vec{\mu}_x}{|\vec{F}_x| \times |\vec{\mu}_x|}\right)$$

Where,

$$\vec{F}_x = (b_1-a_1)\hat{\imath} + (b_2-a_2)\hat{\jmath} + (b_3-a_3)\hat{k}$$

$$\vec{F}_x \cdot \vec{\mu}_x = \mu_1(b_1 - a_1) + \mu_2(b_2 - a_2) + \mu_3(b_3 - a_3)$$

$$\left|\vec{F}_{x}\right| = \sqrt{(b_1 - a_1)^2 + (b_2 - a_2)^2 + (b_3 - a_3)^2}$$

$$|\vec{\mu}_{x}| = \sqrt{\mu_1^2 + \mu_2^2 + \mu_3^2}$$

S.6. Understanding the Origin of the Reaction Barrier in TauD Enzyme

In our study, we observed a reaction barrier of 27 kcal/mol for hydrogen abstraction in the TauD enzyme, which may seem somewhat higher than usual. While our computed barrier is slightly higher than values reported in earlier TauD studies[26], it remains within the range typically observed for high-spin iron–oxo species[27,28]. Nevertheless, we sought to investigate the origin of this relatively elevated barrier. First, we examined the electronic progression of the reaction and found that it proceeds through the usual high-spin σ-channel (see Table S1 for spin density data).

Table S1: Spin densities of the Fe and O atoms in the iron-oxo moiety, and the substrate carbon atom involved in H-abstraction, for all three reactive species.

	**RC**	**TS**	**PC**
**Fe**	3.20	4.17	4.27
**O**	0.56	-0.24	0.19
**Substrate (C)**	0.00	-0.37	-0.96

We then suspected that second-sphere coordination, particularly involving catalytic water molecules, plays a significant role. Interestingly, we have observed that most previous studies on the TauD enzyme do not include active-site water molecules in the QM zone. In contrast, our model incorporates all catalytic water molecules in the QM zone, as they form an organized water channel within the active site. To assess the effect of these water molecules on reactivity, we excluded them from the QM zone and treated them in the MM zone instead. Upon recalculating the barrier with this setup, it decreased significantly to approximately **18.9** kcal/mol, with the reaction becoming exothermic by **3.5** kcal/mol. In this case, we also observed a transition state (TS) with an O–H bond distance of 1.30 Å (see Figure S1), indicating an earlier TS compared to the one observed in the presence of catalytic water molecules. However, the kinetic isotope effect (KIE) calculated from this newly optimized structure was relatively low (KIE = **16**), much lower than both the experimentally observed value and our previously calculated result. This observation suggests a

delicate balance between observed reactivity and the tunneling contribution, both of which appear to be influenced by active-site residues, including catalytic water molecules.

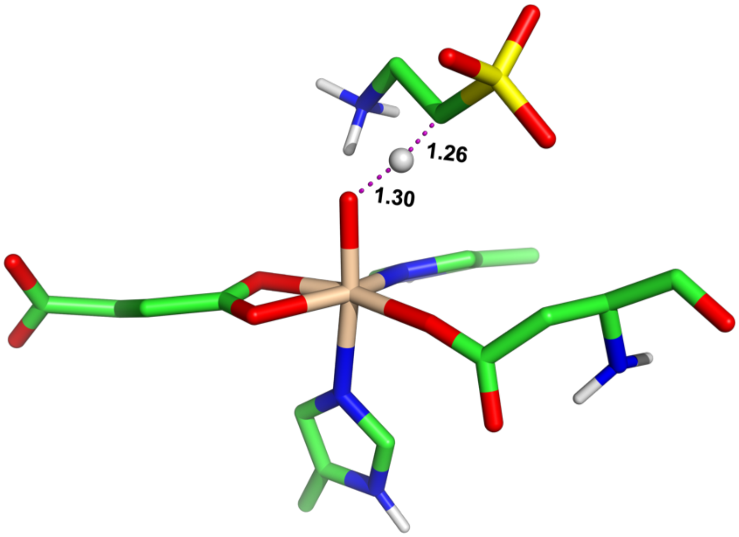

Figure S1: Transition state structure of the TauD enzyme without the catalytic water molecules included in the QM zone. All distances are given in Å.

S.7. Calculation of de Broglie Wavelength of a Hydrogen Atom at Temperature 300 K

Using the equipartition theorem, we can calculate the average kinetic energy associated with a hydrogen atom which can move in all three directions in space.

Average kinetic energy of a hydrogen atom, $< \kappa > = (3/2) \times k_B \times T$

Here, k_B is the Boltzmann constant and T is the temperature, which is 300 K in our case.

Therefore,

$< \kappa > = (3/2) \times k_B \times T$

$= (3/2) \times 1.38 \times 10^{-23} \times 300$ [(J/K) × K]

$= 6.213 \times 10^{-21}$ J

Now, kinetic energy can be expressed as $P^2/2m$, where P and m, are respectively the momentum and the mass of a hydrogen atom (1.674×10^{-27} kg). Therefore, we can write,

$P = \sqrt{\{2m < \kappa >\}}$

$= \sqrt{\{(2 \times 1.674 \times 10^{-27} \times 6.213 \times 10^{-21})}$ [kg × J or kg × kg m^2 s^{-2}]} {J = kg m^2 s^{-2}}

$= \sqrt{\{20.8 \times 10^{-48}\}}$ kg (m/s)

$= 4.56 \times 10^{-24}$ kg (m/s)

Now, using the de Broglie relation,

$\lambda = (h/P)$; h is the Planck's constant and P is the momentum.

$= (6.626 \times 10^{-34}) / (4.56 \times 10^{-24})$ [kg m^2 s^{-1} / kg m s^{-1}]

$= 1.453 \times 10^{-10}$ m

= 1.453 Å

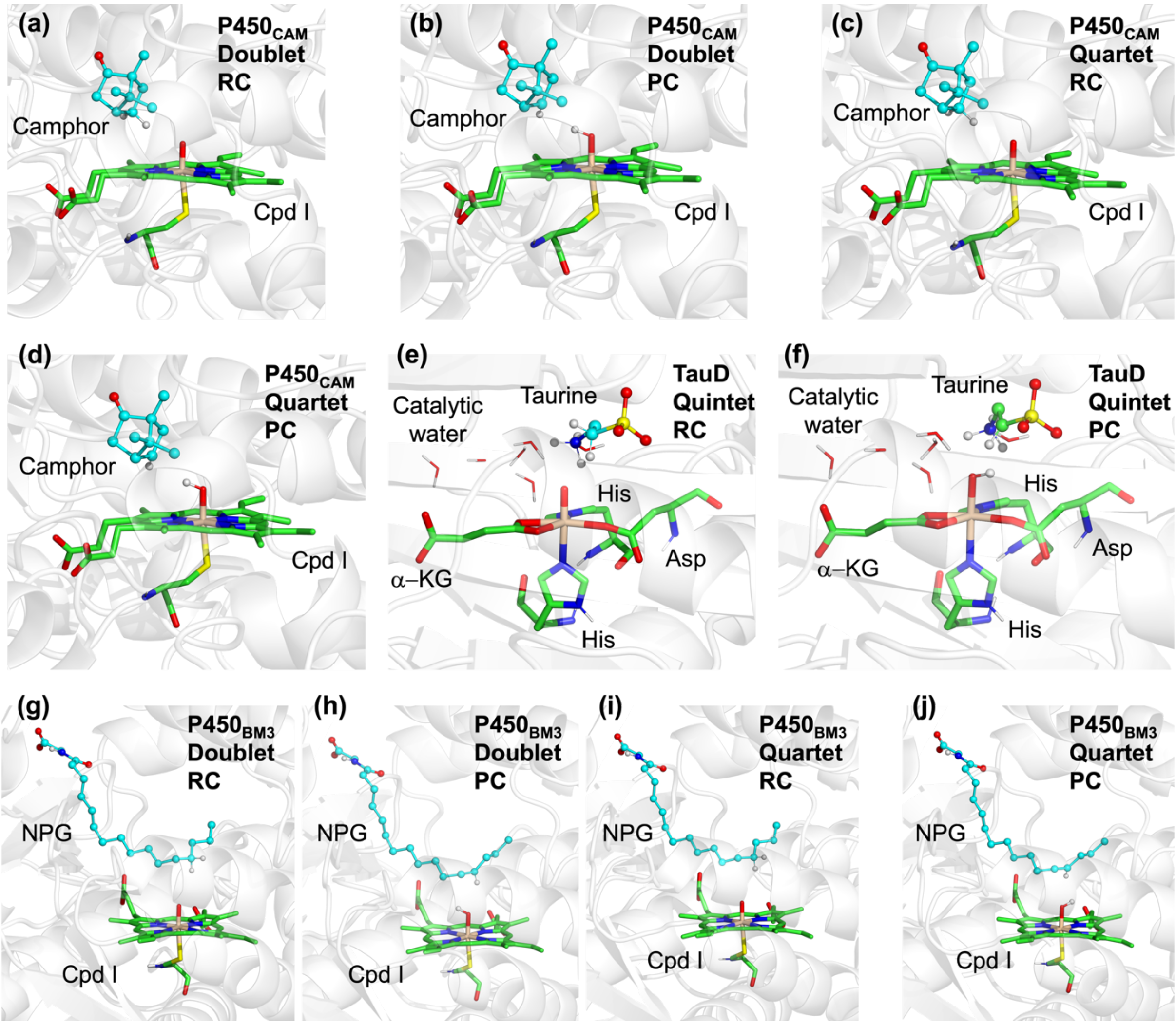

Figure S2: Ball-and-stick representation of the QM zone in the QM/MM-optimized reactant and product complexes (RC and PC) of all three enzymes. The enzyme folds are shown as transparent white cartoons. Here, Cpd I and NPG refer to Compound I and N-Palmitoylglycine, respectively.

Table S2: Eyring-based KIE values obtained from QM/MM calculations in the enzyme environment.

	KIE ($P450_{CAM}$)	KIE ($P450_{BM3}$)	KIE (TauD)
	QM/MM	QM/MM	QM/MM
Doublet	1.33	1.35	NA
Quartet	1.34	1.32	
Quintet	NA		1.35

References:

1. Izaguirre, J. A.; Catarello, D. P.; Wozniak, J. M.; Skeel, R. D. Langevin stabilization of molecular dynamics. *J. Chem. Phys.* **2001**, *114*, 2090-2098.

2. Berendsen, H. J. C.; Postma, J. P. M.; van Gunsteren, W. F.; DiNola, A.; Haak, J. R. Molecular dynamics with coupling to an external bath. *J. Chem. Phys.* **1984**, *81*, 3684-3690.

3. Åqvist, J.; Wennerström; Nervall, M.; Bjelic, S.; Brandsdal, B. O. Molecular dynamics simulations of water and biomolecules with a Monte Carlo constant pressure algorithm. *Chem. Phys. Lett.* **2004**, *384*, 288-294.

4. Ryckaert, J.-P.; Ciccotti, G.; Berendsen, H. J. C. Numerical integration of the cartesian equations of motion of a system with constraints molecular dynamics of n-alkanes. *J. Comput. Phys.* **1977**, *23*, 327-341.

5. Darden, T.; York, D.; Pedersen, L. Particle mesh Ewald: an N.log(N) method for Ewald sums in large systems. *J. Chem. Phys.* **1993**, *98*, 10089-10092.

6. Case, D. A.; Aktulga, H. M.; Belfon, K. et al. *Amber*; University of California: San Francisco, **2023**.

7. Case, D. A.; Aktulga, H. M.; Belfon, K. et al. Amber tools. *J. Chem. Inf. Model.* **2023**, *63*, 6183-6191.

8. Humphrey, W.; Dalke, A.; Schulten, K. VMD - Visual molecular dynamics. *J. Mol. Graphics* **1996**, *14*, 33-38.

9. The PyMOL molecular graphics system, Version 2.0 Schrödinger, LLC.

10. Shaik, S.; Cohen, S.; Wang, Y.; Chen, H.; Kumar, D.; Thiel, W. P450 enzymes: their structure, reactivity, and selectivity—modeled by QM/MM calculations. *Chem. Rev.* **2010**, *110*, 949–1017.

11. Dubey, K. D.; Wang, B.; Shaik, S. Molecular dynamics and QM/MM calculations predict the substrate-induced gating of cytochrome P450 BM3 and the regio- and stereoselectivity of fatty acid hydroxylation. *J. Am. Chem. Soc.* **2016**, *138*, 837–845.

12. Usharani, D.; Janardanan, D.; Shaik, S. Does the TauD enzyme always hydroxylate alkanes, while an analogous synthetic non-heme reagent always desaturates them? *J. Am. Chem. Soc.* **2011**, *133*, 176–179.

13. Ali, H. S.; de Visser, S. P. Electrostatic perturbations in the substrate-binding pocket of taurine/α-ketoglutarate dioxygenase determine its selectivity. *Chem. Eur. J.* **2022**, *28*, e202104167-82.

14. Cao, Y.; Hay, S.; de Visser, S. P. An active site Tyr residue guides the regioselectivity of lysine hydroxylation by nonheme iron lysine-4-hydroxylase enzymes through proton-coupled electron transfer. *J. Am. Chem. Soc.* **2024**, *146*, 11726–11739.

15. Sherwood, P.; de Vries, A. H.; Guest, M. F.; Schreckenbach, G.; Catlow, C. R. A.; French, S. A.; Sokol, A. A.; Bromley, S. T.; Thiel, W.; Turner, A. J.; Billeter, S.; Terstegen, F.; Thiel, S.; Kendrick, J.; Rogers, S. C.; Casci, J.; Watson, M.; King, F.; Karlsen, E.; Sjøvoll, M.; Fahmi, A.; Schäfer, A.; Lennartz, C. QUASI: a general purpose implementation of the QM/MM approach and its application to problem in catalysis. *J. Mol. Struct.: THEOCHEM* **2003**, *632*, 1−28.

16. Metz, S.; Kästner, J.; Sokol, A. A.; Keal, T. W.; Sherwood, P. ChemShell-a modular software package for QM/MM simulations. *Comput. Mol. Sci.* **2014**, *4*, 101−110.

17. Eckart, C. The penetration of a potential barrier by electrons. *Phys. Rev.* **1930**, *35*, 1303−1309.

18. Maity, D. K.; Bell, R. L.; Truong, T. N. Mechanism and quantum mechanical tunneling effects on inner hydrogen atom transfer in free base porphyrin: a direct ab initio dynamics study. *J. Am. Chem. Soc.* **2000**, *122*, 897-906.

19. Zhang, F.; Dibble, T. S. Impact of tunneling on hydrogen-migration of the n-Propylperoxy radical. *Phys. Chem. Chem. Phys.* **2011**, *13*, 17969-17977.

20. Vandeputte, A. G.; Sabbe, K. M.; Reyniers, M. F.; Van Speybroeck, V.; Waroquier, M.; Marin, G. B. Theoretical study of the thermodynamics and kinetics of hydrogen abstractions from hydrocarbons. *J. Phys. Chem. A* **2007**, *111*, 11771-11786.

21. Mandal, D.; Shaik, S. Interplay of Tunneling, Two-state reactivity, and Bell-Evans-Polanyi effects in C-H activation by nonheme Fe(IV)O oxidants. *J. Am. Chem. Soc.* **2016**, *138*, 2094-2097.

22. Mallick, D.; Shaik, S. Kinetic isotope effect probes the reactive-spin state, as well as the geometric feature and constitution of the transition state during H-abstraction by heme compound II complexes. *J. Am. Chem. Soc.* **2017**, *139*, 11451-11459.

23. Mandal, D.; Mallick, D.; Shaik, S. Kinetic isotope effect determination probes the spin of the transition state, its stereochemistry, and its ligand sphere in hydrogen abstraction reactions of oxoiron(IV) complexes. *Acc. Chem. Res.* **2018**, *51*, *1*, 107-117.

24. Kwon, Y. H.; Mai, B. K.; Lee, Y.-M.; Dhuri, S. N.; Mandal, D.; Cho, K.-B.; Kim, Y.; Shaik, S.; Nam, W. Determination of spin inversion probability, H-tunneling correction, and regioselectivity in the two-state reactivity of nonheme iron(IV)-oxo complexes. *J. Phys. Chem. Lett.* **2015**, *6*, 1472-1476.

25. Jónsson, H.; Mills, G.; Jacobsen, K. W. Nudged elastic band method for finding minimum energy paths of transitions. In classical and quantum dynamics in condensed phase simulations; *World Scientific* **1998**; pp 385-404.

26. Álvarez-Barcia, S.; Kästner, J. Atom tunneling in the hydroxylation process of taurine/α-ketoglutarate dioxygenase identified by quantum mechanics/ molecular mechanics simulations. *J. Phys. Chem. B* **2017**, *121*, 5347−5354.

27. Krishnan, A.; Waheed, S. O.; Varghese, A.; Cherilakkudy, F. H.; Schofield, C. J.; Karabencheva-Christova, T. G. Unusual catalytic strategy by non-heme Fe(II)/2- oxoglutarate-dependent aspartyl hydroxylase AspH. *Chem. Sci.* **2024**, *15*, 3466–3484.

28. Waheed, S. O.; Ramanan, R.; Chaturvedi, S. S.; Lehnert, N.; Schofield, C. J.; Christov, C. Z.; Karabencheva-Christova, T. G. Role of structural dynamics in selectivity and mechanism of nonheme Fe(II) and 2-Oxoglutarate-dependent oxygenases involved in DNA repair. *ACS Cent. Sci.* **2020**, *6*, 795−814.

QM coordinates of the QM/MM optimized species

$P450_{CAM}$ – Doublet – RC

S 37.2638007 44.2574464 35.9339944

N 39.8423454 45.7007448 36.4560340

C 40.8077112 44.9624228 35.8493452

C 39.9478466 46.9696903 35.9424180

C	41.5258139	45.7593439	34.8678812
C	41.0323869	47.0396968	34.9609217
C	39.0671310	48.0004417	36.2774873
H	39.2411542	48.9760499	35.8214618
C	37.9421501	47.9411438	37.1111895
N	37.5790937	46.8469813	37.8420547
C	36.4468434	47.2085427	38.5303669
C	36.0466709	48.5633787	38.1801931
C	36.9973896	49.0321591	37.3065087
C	35.8346247	46.4059517	39.4929461
H	34.9501457	46.8124589	39.9806399
C	36.2458960	45.1345708	39.9072008
C	35.6346583	44.3657733	40.9745917
C	37.3012186	43.2180182	39.9840410
N	37.2682719	44.4261276	39.3355510
C	36.2934712	43.1658062	41.0239360
C	38.1282777	42.1561053	39.6290959
H	38.0091336	41.2388908	40.2075283
C	39.0924160	42.1303939	38.6169519
C	39.8884248	40.9609746	38.2601730
N	39.4525252	43.2182784	37.8854239
C	40.4975687	42.8014293	37.1013222
C	41.1214006	43.6319639	36.1764510
H	41.9768264	43.2201575	35.6442732
Fe	38.6164849	45.0883109	37.9635928
C	40.8212236	41.3858492	37.3455188
O	39.6324176	45.6391392	39.1012346
C	39.6613042	47.6952793	43.8807035
C	38.7182594	48.8131055	44.3415017
O	39.0153780	49.7938614	44.9942521
C	37.3462924	48.5140471	43.7474028
C	37.6413768	47.2730551	42.8882244
C	38.5096655	47.7414583	41.6968577
C	39.8739198	48.0686730	42.3711581
C	38.6612739	46.4836860	43.7746107
C	38.0910466	46.0370544	45.1337941
C	39.2451857	45.2445270	43.0780521
C	40.9357447	47.5419658	44.6917298
H	36.6226392	48.3317119	44.5566150
H	36.9782442	49.3876856	43.1911656
H	36.7444911	46.7020269	42.6055141
H	38.0631869	48.6125706	41.1931566
H	38.6302445	46.9604597	40.9337478
H	40.1806517	49.1192293	42.2552803
H	40.6879324	47.4545380	41.9567244
H	37.7377676	46.8644425	45.7653939
H	38.8487328	45.4853936	45.7122197
H	37.2364004	45.3561909	44.9862612
H	39.9983739	44.7578956	43.7163597
H	38.4438103	44.5092415	42.8963447
H	39.7156589	45.4542239	42.1079666
H	40.7378067	47.0810414	45.6732805
H	41.3936705	48.5270039	44.8732794
H	41.6725281	46.9115081	44.1699166
H	36.0737532	43.7666143	36.4312048
H	42.2202913	45.3330346	34.1439830
H	41.3400894	47.9564032	34.4578951
H	35.1717710	49.1114549	38.5298068
H	37.0700759	50.0257846	36.8643636
H	34.7820522	44.6372895	41.5970134

H	36.0758917	42.3272721	41.6854395
H	39.6403361	39.9563167	38.6024912
H	41.6542554	40.8585865	36.8806327

P450$_{CAM}$ – Doublet – TS

S	37.3176472	44.5056234	36.1526080
N	39.8124956	46.0391690	36.6712874
C	40.7528990	45.2901228	36.0372821
C	39.9167799	47.3035206	36.1536092
C	41.4556554	46.0800140	35.0372450
C	40.9840480	47.3664403	35.1513361
C	39.0295698	48.3314569	36.4796182
H	39.2042914	49.3064361	36.0234460
C	37.8796162	48.2602027	37.2765815
N	37.4966888	47.1656585	38.0020917
C	36.3191461	47.5086572	38.6203984
C	35.9234765	48.8550994	38.2449179
C	36.9081826	49.3356641	37.4169042
C	35.6615681	46.6929454	39.5415360
H	34.7388942	47.0751137	39.9734244
C	36.0878538	45.4389322	39.9837435
C	35.4592035	44.6557621	41.0286831
C	37.2507257	43.5870069	40.1697613
N	37.1885908	44.7815612	39.4957974
C	36.1850454	43.5001136	41.1468009
C	38.1415912	42.5537169	39.8897181
H	38.0322737	41.6554019	40.4983229
C	39.1238308	42.5158098	38.8971827
C	39.9245464	41.3423256	38.5548505
N	39.4564108	43.5858234	38.1295559
C	40.4723765	43.1504574	37.3172288
C	41.0640427	43.9591554	36.3540964
H	41.8926276	43.5361802	35.7888582
Fe	38.5238076	45.4347269	38.1495420
C	40.8101811	41.7413008	37.5835625
O	39.6299438	46.0562883	39.3520613
C	39.9267808	47.6888325	43.4959060
C	38.8322569	48.7023540	43.8818433
O	38.9593957	49.6714604	44.5991882
C	37.5487927	48.2523286	43.1836865
C	38.0355129	47.0097421	42.3963888
C	38.9162069	47.5677310	41.2744217
C	40.2051801	48.0060967	41.9818657
C	39.0631467	46.3764488	43.3871035
C	38.4520528	45.9503933	44.7368199
C	39.8014396	45.1697646	42.7927362
C	41.1370001	47.7003932	44.4119295
H	36.7828734	48.0107056	43.9390739
H	37.1318498	49.0542192	42.5602996
H	37.2218309	46.3456478	42.0833694
H	38.4364179	48.3195939	40.6314054
H	39.2188149	46.6911105	40.3023446
H	40.4545235	49.0642941	41.8018576
H	41.0777899	47.4200112	41.6465882
H	38.0690935	46.7849869	45.3432127
H	39.2007996	45.4237854	45.3489889
H	37.6121246	45.2552071	44.5786500
H	40.6577807	44.8897329	43.4214946
H	39.1213596	44.3031620	42.7619301
H	40.1648435	45.3284344	41.7688060

H	40.8931309	47.2670671	45.3954900
H	41.4716323	48.7337111	44.5895937
H	41.9767085	47.1272379	43.9895074
H	36.1359432	43.9560659	36.6063807
H	42.1080838	45.6402454	34.2829188
H	41.2999325	48.2827161	34.6526149
H	35.0235645	49.3862955	38.5548472
H	36.9745455	50.3204188	36.9543879
H	34.5664333	44.8927458	41.6073602
H	35.9797986	42.6606691	41.8110854
H	39.7231456	40.3510493	38.9609000
H	41.6184070	41.2043121	37.0870937

P450$_{CAM}$ – Doublet – PC

S	37.2927367	44.3979752	36.0471338
N	39.7856940	45.8309388	36.4656103
C	40.7694930	45.0836507	35.8767977
C	39.8929443	47.1005741	35.9524359
C	41.4929182	45.8860192	34.9070739
C	40.9961428	47.1663991	34.9946961
C	39.0130420	48.1386781	36.2598168
H	39.1793592	49.1057103	35.7860916
C	37.9020489	48.0781067	37.0987371
N	37.5350908	46.9922350	37.8532810
C	36.3879769	47.3475768	38.5247895
C	35.9943705	48.6960850	38.1552694
C	36.9489661	49.1607427	37.2821744
C	35.7540892	46.5533431	39.4752089
H	34.8563526	46.9511684	39.9420660
C	36.1814632	45.2984323	39.8950355
C	35.5777118	44.5277479	40.9601175
C	37.2837958	43.3883501	40.0173606
N	37.2339463	44.5923604	39.3517163
C	36.2615639	43.3414108	41.0374805
C	38.1229736	42.3268626	39.7037842
H	38.0037805	41.4213429	40.2982374
C	39.0954181	42.3069307	38.7108834
C	39.9069639	41.1454035	38.3706737
N	39.4459169	43.3837959	37.9480150
C	40.4804132	42.9636189	37.1542936
C	41.0936588	43.7678615	36.2013245
H	41.9441140	43.3523198	35.6666673
Fe	38.5376919	45.2220711	37.9522881
C	40.8198037	41.5586229	37.4297917
O	39.6352901	45.8585753	39.1834245
C	39.7418658	47.7000715	43.7794795
C	38.7714256	48.8140551	44.2166782
O	39.0356564	49.7791544	44.9015655
C	37.4201016	48.5161647	43.5724201
C	37.7537628	47.2617028	42.7172694
C	38.6688411	47.7602252	41.6304691
C	39.9993411	48.0573572	42.2716330
C	38.7449574	46.4847717	43.6540698
C	38.1334166	46.0585770	45.0011765
C	39.3406031	45.2380765	42.9833051
C	40.9878217	47.5545590	44.6341916
H	36.6640963	48.3176323	44.3490260
H	37.0667053	49.3871972	43.0047702
H	36.8697683	46.7009027	42.3858495
H	38.3081373	48.2631690	40.7275484

H	39.1774902	46.3774757	39.8822921
H	40.3321534	49.1005709	42.1280811
H	40.8168395	47.4261103	41.8732284
H	37.7877151	46.8952800	45.6252279
H	38.8676337	45.4918010	45.5948625
H	37.2672136	45.3969250	44.8378344
H	40.1253075	44.7890819	43.6106302
H	38.5520149	44.4806948	42.8466773
H	39.7703640	45.4300940	41.9902714
H	40.7546335	47.0964760	45.6087783
H	41.4347639	48.5414801	44.8304346
H	41.7441927	46.9246645	44.1408792
H	36.1066731	43.8700222	36.5149652
H	42.1931476	45.4611388	34.1878920
H	41.3195801	48.0845059	34.5042521
H	35.1163489	49.2439757	38.4972650
H	37.0146990	50.1489785	36.8270519
H	34.7126353	44.7868798	41.5704948
H	36.0483949	42.5087548	41.7077865
H	39.6883602	40.1472076	38.7499949
H	41.6500177	41.0286375	36.9629647

P450$_{CAM}$ – Quartet – RC

S	37.2638759	44.2692822	35.9304041
N	39.8454646	45.7088372	36.4394240
C	40.8095713	44.9725070	35.8275190
C	39.9510084	46.9796981	35.9317324
C	41.5266297	45.7735730	34.8490020
C	41.0341875	47.0539856	34.9492952
C	39.0708102	48.0096072	36.2732081
H	39.2437944	48.9870001	35.8205936
C	37.9474186	47.9469038	37.1080292
N	37.5856188	46.8496248	37.8359599
C	36.4535353	47.2077274	38.5258527
C	36.0515149	48.5633112	38.1801373
C	37.0017369	49.0362359	37.3081346
C	35.8436333	46.4014687	39.4874742
H	34.9596018	46.8057126	39.9778724
C	36.2571907	45.1298005	39.8976570
C	35.6494532	44.3572035	40.9645776
C	37.3140908	43.2130307	39.9657031
N	37.2788457	44.4235468	39.3212258
C	36.3085862	43.1573454	41.0081607
C	38.1413036	42.1529830	39.6053423
H	38.0241403	41.2339965	40.1813083
C	39.1048704	42.1312928	38.5920107
C	39.9053692	40.9651080	38.2337617
N	39.4605685	43.2208956	37.8609636
C	40.5059080	42.8081953	37.0750807
C	41.1255530	43.6416672	36.1500277
H	41.9805797	43.2326616	35.6150701
Fe	38.6185234	45.0895607	37.9433527
C	40.8360492	41.3939974	37.3186311
O	39.6350282	45.6312732	39.0868068
C	39.6621226	47.6952566	43.8794123
C	38.7176176	48.8123812	44.3395432
O	39.0129626	49.7918322	44.9941425
C	37.3468008	48.5128258	43.7422596
C	37.6441206	47.2720370	42.8834098
C	38.5143494	47.7408493	41.6934839
C	39.8772114	48.0692958	42.3702111

C	38.6630527	46.4831202	43.7713840
C	38.0908783	46.0361381	45.1296792
C	39.2490116	45.2443331	43.0757195
C	40.9351912	47.5425042	44.6926062
H	36.6213653	48.3307474	44.5499218
H	36.9798747	49.3864089	43.1852925
H	36.7483106	46.7002026	42.5989693
H	38.0684956	48.6112901	41.1879693
H	38.6365966	46.9592859	40.9312099
H	40.1828564	49.1203056	42.2552890
H	40.6928325	47.4562985	41.9570594
H	37.7354291	46.8631851	45.7605285
H	38.8484385	45.4858278	45.7095230
H	37.2373606	45.3541896	44.9807422
H	40.0008364	44.7576472	43.7155351
H	38.4485685	44.5086611	42.8915859
H	39.7218350	45.4546339	42.1069319
H	40.7355237	47.0826696	45.6742954
H	41.3928500	48.5276219	44.8740969
H	41.6726569	46.9115588	44.1723587
H	36.0739218	43.7777688	36.4271647
H	42.2189604	45.3506958	34.1210458
H	41.3423586	47.9731175	34.4510039
H	35.1761101	49.1096288	38.5312363
H	37.0734109	50.0314521	36.8694154
H	34.7992078	44.6278104	41.5906141
H	36.0920575	42.3165979	41.6671950
H	39.6634301	39.9596441	38.5781088
H	41.6726957	40.8714618	36.8549002

P450$_{CAM}$ – Quartet – TS

S	37.3057368	44.5460722	36.1833709
N	39.8407376	45.9438002	36.6819298
C	40.7779984	45.1914220	36.0289156
C	39.9413207	47.2203698	36.1651560
C	41.4663178	45.9901493	35.0294943
C	41.0002518	47.2771141	35.1558878
C	39.0752892	48.2580708	36.4953878
H	39.2579735	49.2342736	36.0467249
C	37.9282048	48.1839237	37.2947472
N	37.5410627	47.0827390	37.9968858
C	36.3625179	47.4095987	38.6203390
C	35.9679332	48.7637037	38.2637805
C	36.9578384	49.2580515	37.4491202
C	35.7016049	46.5874479	39.5313988
H	34.7798053	46.9688308	39.9652118
C	36.1251113	45.3323103	39.9660927
C	35.4993086	44.5584334	41.0193920
C	37.2629321	43.4620301	40.1418989
N	37.2066237	44.6476674	39.4685482
C	36.2090000	43.3904325	41.1307316
C	38.1431043	42.4201280	39.8468677
H	38.0263198	41.5096589	40.4346286
C	39.1232030	42.4087009	38.8650541
C	39.9185622	41.2417811	38.5021632
N	39.4812756	43.4985177	38.1103260
C	40.4932049	43.0619659	37.2923232
C	41.0904605	43.8662099	36.3288642
H	41.9123448	43.4415199	35.7568548
Fe	38.5911091	45.3095513	38.1226831

C	40.8169115	41.6501616	37.5463479
O	39.5157596	45.9453276	39.4325089
C	39.8650751	47.6564190	43.5549223
C	38.7936235	48.6947027	43.9391153
O	38.9422338	49.6615786	44.6552132
C	37.5026161	48.2772416	43.2352711
C	37.9608743	47.0214952	42.4496370
C	38.8582008	47.5591298	41.3310527
C	40.1510799	47.9755545	42.0432402
C	38.9710705	46.3640551	43.4429014
C	38.3434545	45.9506617	44.7886708
C	39.6820677	45.1400190	42.8511048
C	41.0804334	47.6377394	44.4640681
H	36.7261173	48.0558644	43.9858164
H	37.1114662	49.0901918	42.6097215
H	37.1341256	46.3771537	42.1293242
H	38.3943801	48.3160793	40.6800972
H	39.1245597	46.6772081	40.3858238
H	40.4140289	49.0314435	41.8683303
H	41.0186731	47.3819029	41.7083309
H	37.9601111	46.7913364	45.3859535
H	39.0818053	45.4217786	45.4113780
H	37.5003706	45.2604469	44.6248829
H	40.4950272	44.8067935	43.5108167
H	38.9662509	44.3067167	42.7649811
H	40.1014201	45.3042027	41.8496525
H	40.8349052	47.2034795	45.4466730
H	41.4402508	48.6626308	44.6424595
H	41.9040597	47.0488063	44.0317827
H	36.1281170	43.9671624	36.6104802
H	42.1125463	45.5564148	34.2663886
H	41.3127930	48.1919667	34.6524696
H	35.0686998	49.2944930	38.5763651
H	37.0286752	50.2489380	37.0005746
H	34.6194623	44.8111504	41.6110606
H	35.9984333	42.5578416	41.8019404
H	39.6973345	40.2421155	38.8760537
H	41.6282595	41.1138795	37.0542268

P450$_{CAM}$ – Quartet – PC

S	37.2795483	44.3610868	36.0247086
N	39.7835760	45.7783589	36.4408697
C	40.7714438	45.0332856	35.8551925
C	39.8906660	47.0498299	35.9295870
C	41.4950183	45.8375119	34.8875943
C	40.9950176	47.1167323	34.9733441
C	39.0124726	48.0883540	36.2392088
H	39.1801813	49.0561216	35.7674887
C	37.9045466	48.0292441	37.0827499
N	37.5406067	46.9440823	37.8387330
C	36.3978218	47.3006506	38.5177512
C	36.0036448	48.6498137	38.1506581
C	36.9538118	49.1129413	37.2717618
C	35.7683235	46.5069313	39.4715176
H	34.8751056	46.9073840	39.9450381
C	36.1928778	45.2490169	39.8859410
C	35.5930070	44.4794425	40.9541673
C	37.2814001	43.3303582	39.9926636
N	37.2349155	44.5363460	39.3317508
C	36.2677090	43.2873118	41.0218378

C	38.1112854	42.2651877	39.6663609
H	37.9897881	41.3553173	40.2534073
C	39.0821437	42.2481821	38.6724617
C	39.8910950	41.0866252	38.3279494
N	39.4398924	43.3290782	37.9167348
C	40.4783097	42.9109226	37.1268582
C	41.0965720	43.7178799	36.1794869
H	41.9504607	43.3041926	35.6489908
Fe	38.5390803	45.1661906	37.9253925
C	40.8126255	41.5040669	37.3973293
O	39.6379684	45.7984316	39.1656972
C	39.7372549	47.7138562	43.8128182
C	38.7721215	48.8301465	44.2540027
O	39.0449609	49.7981986	44.9319261
C	37.4153689	48.5353210	43.6200712
C	37.7430061	47.2850200	42.7587944
C	38.6572887	47.7800311	41.6674141
C	39.9905847	48.0758147	42.3055816
C	38.7363916	46.5015312	43.6876858
C	38.1311997	46.0714821	45.0363916
C	39.3244966	45.2557311	43.0092117
C	40.9870114	47.5636850	44.6614203
H	36.6656682	48.3371940	44.4029313
H	37.0590295	49.4085886	43.0573257
H	36.8568868	46.7252243	42.4299939
H	38.2957742	48.2801378	40.7630693
H	39.1748496	46.3353858	39.8410460
H	40.3233136	49.1198960	42.1685052
H	40.8081716	47.4462789	41.9050715
H	37.7864173	46.9069032	45.6624014
H	38.8687739	45.5052210	45.6264576
H	37.2654193	45.4085635	44.8755139
H	40.1081017	44.7988749	43.6323245
H	38.5318980	44.5030067	42.8692123
H	39.7535130	45.4523287	42.0167879
H	40.7598388	47.0992509	45.6344555
H	41.4334257	48.5503102	44.8608093
H	41.7421467	46.9382583	44.1604411
H	36.0907904	43.8411617	36.4946871
H	42.1990912	45.4142231	34.1712319
H	41.3164985	48.0344167	34.4808277
H	35.1288981	49.2001260	38.4971284
H	37.0194748	50.1017758	36.8179315
H	34.7337777	44.7430815	41.5708411
H	36.0522784	42.4551439	41.6920269
H	39.6632903	40.0855172	38.6939479
H	41.6461580	40.9750336	36.9353611

$P450_{BM3}$ – Doublet – RC

S	42.0097560	35.9741665	45.7136172
N	44.3063718	37.8648276	45.1128072
C	44.2153996	39.1221442	45.6249606
C	45.4385195	37.3139695	45.6549362
C	45.3063620	39.3818422	46.5548630
C	46.0518062	38.2283036	46.6213729
C	45.9919481	36.1161726	45.1975560
H	46.9397215	35.8043094	45.6369760
C	45.5123448	35.2756109	44.1894673
N	44.2971900	35.4163958	43.5797813
C	44.1656269	34.3390433	42.7464375

C	45.3420053	33.4860535	42.8257735
C	46.2094115	34.0966328	43.6959645
C	43.0618841	34.1182896	41.9182409
H	43.0819531	33.1975800	41.3344774
C	41.9499961	34.9477749	41.7570025
C	40.8267080	34.6926243	40.8713778
C	40.5883874	36.6546443	41.9702279
N	41.7795783	36.1447206	42.4090713
C	39.9867390	35.7702691	40.9844389
C	40.0047001	37.8315280	42.4548924
H	39.0251459	38.0736914	42.0421238
C	40.5394213	38.7356560	43.3772613
C	39.9095844	39.9865882	43.8013837
N	41.7862462	38.6096720	43.9203396
C	42.0067670	39.7700486	44.6099003
C	43.1762405	40.0158138	45.3307792
H	43.2840181	41.0100811	45.7541011
Fe	43.0902801	37.0604473	43.6725913
C	40.8451948	40.6740081	44.5369262
O	43.8932698	37.7764935	42.4572042
C	42.5750570	38.3485588	35.4431748
C	42.6130368	38.5262318	36.9642638
C	44.0214376	38.8440395	37.4753817
C	44.0920581	39.0762000	38.9868436
C	45.4531953	39.5434394	39.5283242
C	46.5506848	38.4872968	39.7461681
C	47.2323836	37.9164909	38.4943898
C	48.5425578	37.1880205	38.8146807
C	49.2666633	36.6465985	37.5814621
H	49.5656404	37.4628707	36.9027269
H	50.1759355	36.0873442	37.8555576
H	48.6156071	35.9684694	37.0050052
H	49.2142718	37.8729957	39.3636059
H	48.3303471	36.3635968	39.5191987
H	46.5570578	37.2235406	37.9637610
H	47.4402978	38.7375489	37.7837228
H	47.3401927	38.9551153	40.3616536
H	46.1416717	37.6683956	40.3660711
H	45.8507033	40.3214299	38.8473695
H	45.2614047	40.0182092	40.5073523
H	43.3430363	39.8471079	39.2452474
H	43.7796953	38.1697791	39.5376034
H	44.7033690	38.0286965	37.1785727
H	44.3899672	39.7484592	36.9566958
H	41.9322069	39.3459171	37.2621889
H	42.2360052	37.6199844	37.4790955
H	43.2681142	37.5451522	35.1358002
H	42.9122588	39.2808654	34.9633312
H	45.4868553	40.3318794	47.0577731
H	46.9070237	37.9573902	47.2404657
H	45.5008868	32.5598772	42.2734861
H	47.2469647	33.8292650	43.8961269
H	40.6659052	33.8163916	40.2433444
H	39.0915169	35.9604098	40.3924161
H	38.8697963	40.2416668	43.5968287
H	40.8197742	41.6631259	44.9941645
H	41.0714647	35.0613376	45.2769249
H	41.5856459	38.1057246	35.0556643

$P450_{BM3}$ – Doublet – TS

S	42.1027515	35.9792430	45.6443757
N	44.4187323	37.8388708	45.1954209
C	44.3251408	39.0985226	45.6963598
C	45.5440733	37.2900448	45.7462587
C	45.4079215	39.3624441	46.6381115
C	46.1515587	38.2091533	46.7168404
C	46.1037352	36.0923375	45.2920744
H	47.0406214	35.7786124	45.7531730
C	45.6472836	35.2486551	44.2735643
N	44.4508692	35.3885469	43.6280375
C	44.3249941	34.2948758	42.8152958
C	45.4889527	33.4300526	42.9422040
C	46.3444182	34.0550139	43.8151316
C	43.2439668	34.0747390	41.9551533
H	43.2563874	33.1259865	41.4173963
C	42.1664849	34.9315692	41.7175129
C	41.0408404	34.6641312	40.8323829
C	40.8179983	36.6474648	41.9003096
N	42.0170001	36.1523152	42.3324064
C	40.2019380	35.7448743	40.9389544
C	40.2230074	37.8182444	42.3891530
H	39.2404780	38.0476048	41.9803794
C	40.7323932	38.7248582	43.3205102
C	40.0772530	39.9648815	43.7395141
N	41.9680521	38.6064656	43.8918208
C	42.1534258	39.7558643	44.6064203
C	43.2976067	39.9955189	45.3717206
H	43.3890594	40.9875862	45.8045490
Fe	43.2589887	37.0329341	43.7095173
C	40.9866165	40.6521611	44.5087690

O	44.3572082	37.8831633	42.6110971
C	42.0222518	38.3370410	35.4010985
C	41.7638748	38.5185820	36.8998681
C	43.0249330	38.8953306	37.6780802
C	42.7719121	39.2097695	39.1555408
C	44.0525693	39.4155090	39.9697819
C	44.8718985	38.1417337	40.1553063
C	46.3729148	38.3077965	40.0060837
C	47.1957041	37.0272331	39.8689994
C	48.6911043	37.2823905	39.6747294
H	49.1339336	37.7907180	40.5464734
H	49.2417655	36.3418983	39.5102359
H	48.8708368	37.9169513	38.7915143
H	47.0327994	36.3824420	40.7521966
H	46.8170960	36.4729690	38.9946046
H	46.5418244	38.9253043	39.1011910
H	46.7602927	38.9202198	40.8360827
H	44.5923307	37.7960603	41.3810702
H	44.4876223	37.2853712	39.5773771
H	44.6749595	40.1839551	39.4749782
H	43.8030499	39.8240418	40.9651621
H	42.1536349	40.1210667	39.2254301
H	42.1777897	38.3968896	39.6158529
H	43.7535878	38.0677382	37.5884243
H	43.5064549	39.7670515	37.1996549
H	41.0044929	39.3091396	37.0463939
H	41.3333338	37.5997072	37.3447548
H	42.8546108	37.6290669	35.2351793
H	42.3152811	39.3037812	34.9619426
H	45.5824934	40.3155344	47.1373216

H	47.0009542	37.9405157	47.3448755
H	45.6534297	32.4935442	42.4093189
H	47.3787083	33.7862306	44.0297631
H	40.8705391	33.7723211	40.2292748
H	39.2820423	35.9101014	40.3780987
H	39.0423126	40.2137723	43.5049530
H	40.9397604	41.6359352	44.9757381
H	41.1586878	35.0796810	45.1928458
H	41.1498809	37.9596091	34.8676370

$P450_{BM3}$ – Doublet – PC

S	42.0180764	35.9404792	45.7323585
N	44.3260969	37.8041169	45.1525770
C	44.2321026	39.0644782	45.6483490
C	45.4684630	37.2724459	45.6867056
C	45.3353223	39.3487475	46.5622056
C	46.0912400	38.2041579	46.6338775
C	46.0177190	36.0703532	45.2376587
H	46.9715688	35.7645997	45.6689699
C	45.5286747	35.2166036	44.2408450
N	44.3083424	35.3456876	43.6455506
C	44.1798877	34.2694193	42.8087453
C	45.3636985	33.4254785	42.8794029
C	46.2334698	34.0449314	43.7415223
C	43.0824878	34.0502832	41.9700825
H	43.1069519	33.1252857	41.3929868
C	41.9747222	34.8845110	41.7816168
C	40.8585556	34.6237218	40.8872306
C	40.6054385	36.5820145	41.9892145
N	41.8025407	36.0838255	42.4274962
C	40.0107364	35.6965641	41.0002669
C	40.0119937	37.7541207	42.4810313
H	39.0337056	37.9935324	42.0635872
C	40.5372160	38.6594825	43.4084945
C	39.9152253	39.9212180	43.8111410
N	41.7783306	38.5277118	43.9657811
C	42.0041698	39.6936312	44.6419047
C	43.1797968	39.9449275	45.3556023
H	43.2840012	40.9427511	45.7719200
Fe	43.0641534	36.9475022	43.7896566
C	40.8512769	40.6077039	44.5485734
O	43.9887665	37.7810651	42.4799186
C	42.4827678	38.3360035	35.4890773
C	42.4209945	38.4970458	37.0122825
C	43.7740939	38.8593887	37.6304549
C	43.7225580	39.0779577	39.1433909
C	45.0940806	39.3760854	39.7873352
C	46.0458172	38.2220343	39.7914137
C	47.4471648	38.3172976	39.2837492
C	48.1098019	36.9684477	38.9826076
C	49.5717174	37.0723583	38.5481853
H	50.1841919	37.5517104	39.3295910
H	50.0060240	36.0794076	38.3470969
H	49.6778944	37.6758263	37.6310691
H	48.0369527	36.3327793	39.8840197
H	47.5246967	36.4589374	38.1983085
H	47.4667017	38.9481026	38.3749042
H	48.0828210	38.8594653	40.0222779
H	43.4977487	37.7402585	41.6448724
H	45.7703394	37.3541052	40.4037159

H	45.5433818	40.2392386	39.2691508
H	44.9019714	39.6887898	40.8344392
H	43.0307588	39.9100033	39.3696940
H	43.2793640	38.1799158	39.6204703
H	44.5049578	38.0611030	37.4043235
H	44.1636829	39.7743091	37.1480231
H	41.6865980	39.2834454	37.2708033
H	42.0490017	37.5688660	37.4910891
H	43.2580451	37.5989957	35.2133118
H	42.7595070	39.2984342	35.0299948
H	45.5128489	40.3062781	47.0517836
H	46.9565669	37.9457304	47.2441984
H	45.5255146	32.5000031	42.3267928
H	47.2764544	33.7913545	43.9311247
H	40.7029502	33.7476315	40.2576912
H	39.1134778	35.8796935	40.4091174
H	38.8819169	40.1866823	43.5878258
H	40.8310430	41.6030482	44.9923609
H	41.0655665	35.0411805	45.2983837
H	41.5376787	38.0069386	35.0571166

$P450_{BM3}$ – Quartet – RC

S	42.0019422	35.9770186	45.7217026
N	44.3109590	37.8507306	45.1194319
C	44.2182626	39.1087119	45.6303516
C	45.4440529	37.3027242	45.6610148
C	45.3099189	39.3709996	46.5587207
C	46.0571721	38.2185109	46.6258469
C	45.9989419	36.1058371	45.2023697
H	46.9462974	35.7937366	45.6425131
C	45.5215492	35.2671904	44.1918526
N	44.3071463	35.4081246	43.5792698
C	44.1760156	34.3284495	42.7482906
C	45.3529951	33.4770064	42.8289891
C	46.2194775	34.0887595	43.6992014
C	43.0714229	34.1036953	41.9220556
H	43.0924414	33.1821957	41.3395767
C	41.9577119	34.9307317	41.7621073
C	40.8350629	34.6762176	40.8758809
C	40.5961598	36.6377135	41.9761276
N	41.7867935	36.1265809	42.4159411
C	39.9952237	35.7539683	40.9893782
C	40.0120576	37.8158445	42.4578436
H	39.0344025	38.0585029	42.0408425
C	40.5441596	38.7211965	43.3801729
C	39.9146509	39.9742962	43.7999325
N	41.7887634	38.5941465	43.9275687
C	42.0092590	39.7558147	44.6147252
C	43.1778847	40.0012846	45.3367582
H	43.2850333	40.9955097	45.7603144
Fe	43.0988779	37.0403889	43.6778252
C	40.8487823	40.6615240	44.5370494
O	43.8828071	37.7792609	42.4653629
C	42.5736972	38.3432143	35.4436521
C	42.6112891	38.5206909	36.9647971
C	44.0195035	38.8402462	37.4756924
C	44.0902807	39.0728318	38.9870194
C	45.4513726	39.5407984	39.5280455
C	46.5494273	38.4852484	39.7456562
C	47.2319517	37.9153206	38.4938809

C	48.5423369	37.1873327	38.8144408
C	49.2672151	36.6465197	37.5813816
H	49.5659960	37.4631087	36.9029407
H	50.1766652	36.0876815	37.8557604
H	48.6167253	35.9681432	37.0045766
H	49.2135545	37.8724246	39.3638218
H	48.3301692	36.3626419	39.5186611
H	46.5571684	37.2222754	37.9626915
H	47.4397427	38.7368196	37.7837011
H	47.3383869	38.9531407	40.3617821
H	46.1408599	37.6655259	40.3647971
H	45.8482614	40.3189442	38.8469391
H	45.2596502	40.0156129	40.5070615
H	43.3410255	39.8435341	39.2452964
H	43.7782504	38.1665243	39.5381228
H	44.7022284	38.0255736	37.1788621
H	44.3869389	39.7449499	36.9567068
H	41.9293486	39.3394491	37.2627379
H	42.2353713	37.6139572	37.4795653
H	43.2685144	37.5413622	35.1361873
H	42.9090843	39.2763377	34.9641183
H	45.4902753	40.3218114	47.0602140
H	46.9135547	37.9493115	47.2440764
H	45.5122764	32.5503134	42.2776846
H	47.2572167	33.8225676	43.8999660
H	40.6750149	33.8008498	40.2464502
H	39.0999545	35.9445878	40.3975805
H	38.8760033	40.2306413	43.5912095
H	40.8236354	41.6513971	44.9926653
H	41.0652533	35.0631374	45.2837729
H	41.5849248	38.0983690	35.0557770

$P450_{BM3}$ – Quartet – TS

S	42.1007175	35.9909791	45.6161085
N	44.3743990	37.8247308	45.1213499
C	44.2701466	39.0887011	45.6333267
C	45.5028223	37.2699274	45.6794144
C	45.3536985	39.3445537	46.5714762
C	46.0982904	38.1915289	46.6510346
C	46.0661938	36.0707048	45.2489248
H	47.0025176	35.7591386	45.7104667
C	45.5997134	35.2309186	44.2362907
N	44.4013125	35.3717736	43.5973569
C	44.2621531	34.2792899	42.7828145
C	45.4297161	33.4171593	42.9007389
C	46.2915972	34.0380876	43.7695164
C	43.1746306	34.0440388	41.9403204
H	43.1920190	33.1015660	41.3935106
C	42.0838043	34.8889338	41.7354530
C	40.9640727	34.6295920	40.8420670
C	40.7174999	36.6016733	41.9285498
N	41.9098710	36.0977978	42.3671128
C	40.1190015	35.7057367	40.9519985
C	40.1247740	37.7755350	42.4028593
H	39.1426936	38.0056242	41.9947803
C	40.6585607	38.6880438	43.3073815
C	40.0167362	39.9352142	43.7243153
N	41.9062005	38.5797486	43.8678273
C	42.1017142	39.7339695	44.5768106
C	43.2419728	39.9815518	45.3374381

H	43.3261534	40.9643544	45.7893166
Fe	43.1966587	37.0228593	43.6791683
C	40.9332630	40.6265572	44.4800463
O	44.0686023	37.7577410	42.4204938
C	42.0788622	38.3828252	35.4333103
C	41.8420686	38.5755860	36.9350304
C	43.1185779	38.9563632	37.6854989
C	42.9108558	39.2680774	39.1711618
C	44.2232486	39.4933417	39.9302981
C	45.0449318	38.2236264	40.1071337
C	46.5449596	38.3525666	39.9551840
C	47.3351893	37.0480889	39.8515797
C	48.8320130	37.2622956	39.6220693
H	49.2990172	37.7799086	40.4749015
H	49.3566744	36.3047243	39.4739580
H	49.0117454	37.8703249	38.7202582
H	47.1775922	36.4386024	40.7602767
H	46.9247128	36.4716087	39.0071196
H	46.7059057	38.9316982	39.0222638
H	46.9639591	38.9959152	40.7454162
H	44.6920424	37.8381165	41.2824520
H	44.6478512	37.3626550	39.5418835
H	44.8324819	40.2488878	39.4012951
H	44.0101287	39.9277457	40.9234043
H	42.2831094	40.1702501	39.2659897
H	42.3461921	38.4501247	39.6575271
H	43.8485075	38.1321120	37.5742214
H	43.5845017	39.8304902	37.1958296
H	41.0865616	39.3684955	37.0869109
H	41.4193559	37.6603642	37.3944697
H	42.9142054	37.6798477	35.2613528
H	42.3571038	39.3482796	34.9820229
H	45.5335338	40.2979395	47.0682477
H	46.9448858	37.9256459	47.2840035
H	45.5932446	32.4813403	42.3663530
H	47.3256159	33.7660161	43.9812932
H	40.8000062	33.7447494	40.2270849
H	39.2091822	35.8781854	40.3770571
H	38.9805517	40.1886548	43.5003585
H	40.8922558	41.6145750	44.9385289
H	41.1470921	35.0852242	45.1983118
H	41.2030277	37.9923811	34.9151070

P450$_{BM3}$ – Quartet – PC

S	42.0277674	35.9745989	45.7011841
N	44.2980727	37.7810871	45.1615001
C	44.2056415	39.0574670	45.6455660
C	45.4364114	37.2366874	45.6994954
C	45.3062462	39.3256267	46.5586228
C	46.0494012	38.1710891	46.6451073
C	45.9975606	36.0359509	45.2649359
H	46.9368710	35.7209181	45.7175554
C	45.5297234	35.2151526	44.2407776
N	44.3263983	35.3611843	43.5955422
C	44.1886590	34.2748536	42.7663185
C	45.3637158	33.4271625	42.8676273
C	46.2236752	34.0375396	43.7477786
C	43.0914391	34.0323259	41.9414622
H	43.1156704	33.1052384	41.3703463
C	41.9839600	34.8616932	41.7799947

C	40.8675759	34.6133771	40.8840198
C	40.6014067	36.5692947	41.9969434
N	41.7937006	36.0543668	42.4391588
C	40.0178231	35.6839758	41.0033642
C	40.0090178	37.7418016	42.4633134
H	39.0303899	37.9782898	42.0494359
C	40.5587585	38.6559054	43.3602907
C	39.9382294	39.9141499	43.7702237
N	41.8060194	38.5391749	43.9108760
C	42.0302305	39.7012754	44.6026299
C	43.1783945	39.9502859	45.3492943
H	43.2750864	40.9385630	45.7859892
Fe	43.0695864	36.9521197	43.7417304
C	40.8724382	40.6051325	44.5070541
O	43.9621958	37.7689202	42.4102139
C	42.3901535	38.4031787	35.4899745
C	42.2864714	38.5835147	37.0093309
C	43.6253855	38.9519273	37.6530807
C	43.5448572	39.2299053	39.1564003
C	44.9165845	39.5128735	39.7983072
C	45.8143187	38.3210270	39.8335319
C	47.2574406	38.3823117	39.4658668
C	47.9381330	37.0272602	39.2531499
C	49.4231726	37.1297036	38.9064419
H	49.9844039	37.6201888	39.7179857
H	49.8702709	36.1359859	38.7427600
H	49.5816770	37.7212029	37.9893800
H	47.8112739	36.4134427	40.1639753
H	47.4062889	36.4968096	38.4456523
H	47.3541684	38.9902444	38.5434666
H	47.8237150	38.9630935	40.2272441
H	44.8540761	37.4063159	42.2763058
H	45.3664072	37.3472731	40.0648393
H	45.4146443	40.3403040	39.2660786
H	44.7396595	39.8715108	40.8319652
H	42.8822585	40.0945457	39.3361061
H	43.0777136	38.3772342	39.6826250
H	44.3472823	38.1331906	37.4702649
H	44.0425659	39.8391968	37.1425446
H	41.5492752	39.3767463	37.2358171
H	41.9006345	37.6648438	37.4941529
H	43.1925227	37.6848572	35.2428545
H	42.6497847	39.3674023	35.0248194
H	45.5017717	40.2882843	47.0309865
H	46.9061683	37.9131606	47.2675945
H	45.5295194	32.4990945	42.3205751
H	47.2600879	33.7694319	43.9528013
H	40.7116503	33.7419066	40.2481790
H	39.1213145	35.8679571	40.4113419
H	38.9022209	40.1767473	43.5562236
H	40.8451821	41.5991403	44.9534539
H	41.0832177	35.0597594	45.2825650
H	41.4666041	38.0385244	35.0403626

TauD – Quintet – RC

C	35.3531141	43.7701176	37.8131477
H	35.2246552	43.7413888	36.7203300
H	35.2788151	44.8251589	38.1057392
C	36.6835410	43.2009283	38.1859092
N	37.3238829	43.4909813	39.3714792

H	36.9622781	44.1264202	40.0926642
C	38.4163985	42.7169198	39.4847752
H	39.1072879	42.7307132	40.3217126
N	38.5273346	41.9411314	38.4172120
C	37.4657390	42.2333225	37.5876417
H	37.2888719	41.6843891	36.6665696
N	35.6088333	39.2474125	35.0603268
H	35.7775135	38.7350599	35.9230525
C	36.7645235	39.6739521	34.2782044
H	37.0301910	40.7342884	34.4592988
C	38.0255359	38.8433107	34.6638636
H	37.9640466	37.8637756	34.1663377
H	38.9114893	39.3830467	34.3005683
C	38.1293400	38.5829367	36.1694517
O	38.9225302	39.2985285	36.8489493
O	37.3828537	37.6830579	36.6296110
C	36.5557810	39.4712473	32.7535800
O	35.8432732	38.5655320	32.3625800
C	36.8339495	39.1308118	42.9851001
H	37.5903267	39.2637351	43.7731521
H	36.3523474	38.1619416	43.1806639
C	37.4702539	39.1191733	41.6365581
N	36.9459768	38.3941228	40.5827615
H	36.0978852	37.8416291	40.6108763
C	37.6741313	38.6272084	39.4759575
H	37.4647845	38.1879206	38.5017423
N	38.6695090	39.4556687	39.7598794
C	38.5580468	39.7665370	41.1057882
H	39.2435470	40.4474753	41.5992420
Fe	39.8681726	40.3754533	38.3090740

O	40.8205267	41.1088115	37.2187094
O	41.5178393	39.4127529	39.1429809
C	41.6987353	40.3310475	40.0217849
O	40.8847785	41.2876115	40.0404882
C	42.8854348	40.2796478	40.9479558
C	42.6676185	41.0644742	42.2411350
C	43.9108676	41.1599329	43.1319730
O	44.9551968	41.6581511	42.6268196
O	43.8093274	40.7709171	44.3194414
H	42.3799783	42.0910387	41.9632360
H	41.8422709	40.6374124	42.8268412
H	43.1504836	39.2262972	41.1273974
H	43.7124797	40.7385262	40.3778063
C	39.2769423	44.5817516	34.9969082
C	40.2197902	43.3872272	34.9551391
N	38.8939312	44.9859199	36.3797713
S	40.1668995	42.6257411	33.3034030
O	41.1142308	41.4772510	33.3329733
O	38.7309326	42.2157764	33.1839434
O	40.5532381	43.6904972	32.3420974
H	41.2632737	43.6510723	35.1713329
H	39.9297418	42.5882544	35.6531713
H	38.3091396	44.2488798	36.8064782
H	39.7204409	45.0819353	37.0409476
H	39.7174052	45.4533376	34.4995038
H	38.3455355	44.3292101	34.4742749
H	38.2824383	45.8298197	36.3708517
O	43.7200015	39.9045267	37.4276885
H	43.0119745	39.6201706	38.0306999
H	44.0870088	40.6981248	37.8571758

O	36.5481568	46.5188417	36.9495116
H	35.8252322	46.7411481	36.3198328
H	36.8357010	47.3710454	37.3339455
O	42.7384957	43.0809964	36.9917299
H	42.1890527	42.2694573	36.9858462
H	43.5494729	42.8330337	37.5009628
O	44.5023320	42.3565148	38.8458533
H	45.3943288	42.2012876	39.2859269
H	44.1294632	43.0965570	39.3869400
O	40.9623355	44.7739419	38.1146163
H	40.9235976	44.6139527	39.0884943
H	41.6562523	44.1446035	37.7856041
O	46.5526523	41.9320706	40.4188998
H	45.9890942	41.7417408	41.2011738
H	47.0000691	42.7739852	40.6609575
H	34.5390416	43.2320551	38.2987834
H	36.0518479	39.8819933	43.0951483
H	34.7952203	39.8285553	35.0436165
H	37.0788779	40.1707312	32.1015378

TauD – Quintet – TS

C	35.1540703	43.8359947	37.8594329
H	34.9847601	43.8326933	36.7731429
H	35.0638635	44.8794713	38.1820981
C	36.5120916	43.2979559	38.1660745
N	37.2136500	43.6215363	39.3055225
H	36.8746107	44.2426427	40.0484717
C	38.3302560	42.8782556	39.3668672
H	39.0658691	42.9189190	40.1641141
N	38.3947894	42.0859826	38.3057653
C	37.2730531	42.3331296	37.5440688
H	37.0356436	41.7466715	36.6610672
N	35.6105735	39.3309414	35.0426275
H	35.7861559	38.7991989	35.8919742
C	36.7521025	39.7355968	34.2320958
H	37.0356786	40.7926563	34.4104537
C	37.9960141	38.8704963	34.5888652
H	37.8764386	37.8847435	34.1159764
H	38.8906750	39.3721154	34.1927580
C	38.1241072	38.6547116	36.0957300
O	38.8674334	39.4442500	36.7480210
O	37.4281849	37.7295871	36.5864502
C	36.5250363	39.5342678	32.7149191
O	35.8119436	38.6233673	32.3353570
C	36.9249342	39.1735463	42.9165091
H	37.6828304	39.3206677	43.7011693
H	36.4842726	38.1823163	43.1003882
C	37.5462852	39.2135641	41.5592884
N	37.0632798	38.4480509	40.5160051
H	36.2634771	37.8287847	40.5611133
C	37.7551271	38.7292481	39.3969030
H	37.5657386	38.2662580	38.4301926
N	38.6902803	39.6308454	39.6624166
C	38.5759404	39.9397730	41.0094786
H	39.2172956	40.6721496	41.4882690
Fe	39.7729719	40.6100442	38.1701641
O	40.7491132	41.4541878	36.9617442
O	41.4875761	39.7300179	38.8831456
C	41.6726880	40.5838275	39.8298953
O	40.8552349	41.5282213	39.9318697

C	42.8741373	40.4531857	40.7388963
C	42.6324268	41.0140263	42.1407157
C	43.8763486	41.1332569	43.0350965
O	44.9151962	41.6561801	42.5428422
O	43.7709449	40.7559749	44.2269834
H	42.2312380	42.0334751	42.0192655
H	41.8710083	40.4299953	42.6760101
H	43.1943440	39.4001835	40.7511586
H	43.6726342	41.0328976	40.2440509
C	39.9334429	44.4481709	35.2661292
C	39.6632091	42.9619656	35.1850245
N	39.2412624	45.1746173	36.3748280
S	40.0983888	42.3292754	33.5283410
O	40.6012787	40.9359365	33.6703040
O	38.7815352	42.3916397	32.8189596
O	41.0968411	43.2814109	32.9823259
H	40.2735008	42.1393958	36.1479805
H	38.6215682	42.6846541	35.3813805
H	38.2375536	44.9515838	36.4933358
H	39.7847228	45.0146670	37.2907642
H	41.0111513	44.6410056	35.3744027
H	39.6180099	44.9169923	34.3201947
H	39.2195534	46.1808887	36.1395295
O	43.8698869	39.9148620	37.4477668
H	43.0823093	39.7595979	37.9996664
H	44.2309929	40.7494539	37.7917123
O	36.5872098	46.3005586	36.8980906
H	35.8491375	46.5358953	36.2940694
H	36.8942762	47.1513586	37.2661809
O	42.7414475	43.2147665	37.0610187
H	42.1813572	42.4050259	37.0049071
H	43.5664285	42.9458546	37.5350499
O	44.6078752	42.5172802	38.8047641
H	45.4759033	42.2939262	39.2613940
H	44.2485688	43.2407445	39.3763136
O	40.9527204	44.7185671	38.3212662
H	40.9466022	44.5467846	39.2950778
H	41.6621950	44.1206984	37.9525155
O	46.5973124	41.9475509	40.4093371
H	46.0048827	41.7481482	41.1679020
H	47.0451548	42.7801301	40.6779716
H	34.3780837	43.2604835	38.3641099
H	36.1139431	39.8889572	43.0526993
H	34.7883236	39.8999831	35.0343208
H	37.0446817	40.2274667	32.0534729

TauD – Quintet – PC

C	35.3597443	43.8009426	37.8116391
H	35.2309650	43.7803720	36.7193025
H	35.2815907	44.8545087	38.1086383
C	36.6936982	43.2350050	38.1768751
N	37.3136248	43.4840338	39.3832772
H	36.9400846	44.0961417	40.1177740
C	38.4066070	42.7102713	39.4849605
H	39.0792934	42.6951317	40.3374203
N	38.5431024	41.9727686	38.3925882
C	37.4918145	42.2951941	37.5584711
H	37.3367282	41.7855395	36.6108744
N	35.5940937	39.2475487	35.0512679
H	35.7651723	38.7568613	35.9260397

C	36.7519325	39.6790296	34.2752151
H	37.0262822	40.7348455	34.4689050
C	38.0056093	38.8292295	34.6389679
H	37.9148257	37.8490275	34.1473459
H	38.8902917	39.3520823	34.2484371
C	38.1388349	38.5728307	36.1407671
O	38.9704181	39.2763944	36.7969536
O	37.3893932	37.6949763	36.6260564
C	36.5436847	39.4907699	32.7490343
O	35.8413594	38.5811079	32.3493727
C	36.8224038	39.1310913	43.0057815
H	37.5810445	39.2713000	43.7903320
H	36.3385016	38.1663312	43.2154161
C	37.4578491	39.0945816	41.6576486
N	36.9019810	38.3891771	40.6061863
H	36.0269962	37.8807249	40.6356068
C	37.6449583	38.5862771	39.5010709
H	37.4171853	38.1601523	38.5257557
N	38.6772457	39.3662617	39.7807310
C	38.5762246	39.6871023	41.1241903
H	39.2941697	40.3370439	41.6157003
Fe	39.8978946	40.3578421	38.2632035
O	40.9905046	41.0811054	36.8936774
O	41.6366902	39.3465976	39.0894716
C	41.7821695	40.2945426	39.9278302
O	40.9291775	41.2357348	39.9008176
C	42.9549197	40.3271336	40.8683804
C	42.6642278	41.0359357	42.1914731
C	43.8884546	41.1746336	43.1049991
O	44.9290568	41.6965418	42.6158300
O	43.7763083	40.7928835	44.2943446
H	42.3082433	42.0524512	41.9573227
H	41.8650554	40.5263145	42.7470879
H	43.3139381	39.2967082	41.0115514
H	43.7412499	40.8810481	40.3256816
C	39.8668269	44.8137488	35.0656847
C	40.2150771	43.3755297	34.9654903
N	39.0356343	45.1174219	36.2729370
S	40.1552035	42.5701245	33.4042790
O	41.0239161	41.3585314	33.5084166
O	38.7027396	42.2325416	33.2539349
O	40.6196163	43.5607040	32.3999314
H	40.8439566	40.6415663	36.0425068
H	40.3836155	42.7408353	35.8415089
H	38.2686657	44.4410968	36.3971091
H	39.6558222	45.0430002	37.1438646
H	40.7756172	45.4372638	35.1612752
H	39.3391230	45.1659184	34.1680710
H	38.5736264	46.0423346	36.1953380
O	43.9052945	39.9154820	37.4027870
H	43.1653012	39.6341219	37.9677602
H	44.1976021	40.7512474	37.8093594
O	36.5598732	46.5192487	36.9107590
H	35.8205694	46.7528720	36.3058177
H	36.8245126	47.3599996	37.3321307
O	42.6764053	43.1434164	37.0101697
H	42.1595925	42.3006497	36.9248337
H	43.4931771	42.8989530	37.5028638
O	44.5386007	42.4357888	38.8173908
H	45.4183126	42.2661211	39.2725964

H	44.1582526	43.1659066	39.3652992
O	40.8702463	44.7260895	38.1706294
H	40.8596683	44.5587587	39.1445360
H	41.5866897	44.1255508	37.8152677
O	46.5583238	41.9698997	40.4276960
H	45.9808494	41.7809017	41.2000587
H	47.0100457	42.8062321	40.6788437
H	34.5460201	43.2579199	38.2923120
H	36.0422538	39.8853115	43.1086652
H	34.7869618	39.8377368	35.0375440
H	37.0625842	40.2002858	32.1045208
